# Sensing Platform Technologies of the Transient Electromagnetic Method for Urban Underground Space Detection: Challenges and Advances

**DOI:** 10.3390/s26175339

**Published:** 2026-08-23

**Authors:** Hanlin Guo, Qiyan Gu, Jian Xu, Haotian Shi, Leixiang Bian, Zhan Xu

**Affiliations:** 1 State Key Laboratory of Spatial Datum, Xi’an 710054, China; hl_guo2025@163.com (H.G.); malan_xj@163.com (J.X.); 2 School of Safety Science and Engineering, Nanjing University of Science and Technology, Nanjing 210094, China; m19862318457@163.com (Q.G.); dmu6hao@163.com (H.S.); lxbian@163.com (L.B.)

**Keywords:** urban underground space, transient electromagnetic method (TEM), sensing platform, mobilized profiling, imaging technology

## Abstract

As urban underground spaces and infrastructure development accelerate, subsurface elements such as buried pipelines, integrated utility tunnels, subway tunnels, cavity defects, and deep-seated hidden hazards become increasingly intertwined. Consequently, urban target detection is characterized by pronounced scale discrepancies, intense environmental interference, and severely confined operational spaces. The transient electromagnetic method (TEM) is highly valuable for rapid surveys and hazard identification in urban underground spaces owing to its inherent advantages, including non-contact operation, adaptability to hardened pavements, high sensitivity to low-resistivity anomalies, and the ability to probe a broad range of depths. In recent years, research has shifted from improving isolated instrumentation to synergistically optimizing sensing platforms, transmitter–receiver systems, anti-interference methodologies, and imaging interpretation workflows. Specifically, small-loop configurations and high-frequency excitation technologies have improved shallow-sounding capabilities in confined urban spaces; anti-interference techniques have increased data reliability in complex noise environments; and apparent resistivity mapping, virtual wave-field migration, and rapid inversion methodologies have enabled profiling results to transition from qualitative identification to fine-scale interpretation. Concurrently, the evolution of ground-towed, UAV-borne, helicopter-borne, and semi-airborne platforms has progressively endowed urban TEM profiling with continuous, mobile, and scenario-specific operational capabilities. Looking to the future, further technical breakthroughs in urban TEM technology are required to improve shallow-resolution, deep-seated penetration, multi-source interference decoupling, and real-time concurrent imaging.

## 1. Introduction

The construction of smart cities has become an inevitable trend and a vital catalyst for future economic growth [1]. In the ongoing transition of modern cities towards intelligence and high resilience, the orderly development, safe operation, and maintenance of underground spaces are the lifelines that sustain the evolution of sustainable urban areas. Recent studies have highlighted that urban underground space has expanded beyond traditional infrastructure functions to support transportation, municipal facilities, and public services, increasing the demand for accurate underground characterization technologies [2]. The staggering increase in underground engineering density has made early warnings about abrupt road-collapse hazards and the routine surveying of complex power-cable and drainage-pipe networks more challenging [3]. In recent years, sudden ground collapses triggered by concealed cavities, loosened soil around leaking pipelines, and construction disturbance have repeatedly occurred in densely built-up cities, causing casualties, traffic paralysis, and substantial economic losses; the rapid screening and automatic identification of such subsurface distress has therefore become an urgent public-safety priority [4]. These engineering demands place rigorous requirements on geophysical detection technologies and call for a balance between high resolution and operational flexibility. The transient electromagnetic method (TEM) is highly sensitive to subsurface low-resistivity anomalies and offers significant technical advantages [5], including high vertical resolution and non-contact surface operation [6,7,8]. This makes it well-suited to addressing the challenges of fine-scale exploration of underground urban spaces.

Compared with conventional methods such as electrical resistivity tomography (ERT), gravity exploration, ground-penetrating radar (GPR), and shallow seismic exploration, the transient electromagnetic method offers significant advantages for detecting underground cavities in urban areas. Although ERT offers high resolution, the deployment of electrode arrays makes it inapplicable to urban paved/hardened roads, resulting in low operational efficiency [9,10]. Conversely, while GPR can achieve high-resolution shallow imaging, its high-frequency electromagnetic waves are severely attenuated in highly conductive media (e.g., wet clay and metallic pipelines), limiting its effective detection depth to typically less than 5 m. This fails to meet the requirements for an early warning system for deep-seated hazards [11,12,13]. Shallow seismic exploration relies heavily on active seismic sources and dense geophone arrays, which yield low signal-to-noise ratios (SNRs), pose execution challenges in noisy urban environments, and result in an insensitive response to low-resistivity bodies [14,15]. In contrast, the transient electromagnetic (TEM) method uses ungrounded loop sources, eliminating the need for electrode coupling. It is naturally sensitive to low-resistivity targets, is robustly immune to electromagnetic interference, and can detect targets at depths ranging from several to hundreds of meters. This reconciles shallow, fine-scale imaging with deep hazard identification [16,17]. Quantitatively, GPR equipped with 100–400 MHz antennas rarely exceeds 5–10 m penetration in conductive urban soils [11], ERT production rates on paved roads are constrained by electrode deployment to limited profile mileage per day [9,10], and shallow seismic acquisition under traffic noise suffers from a low SNR [14,15]; in contrast, towed TEM systems acquire continuous profiles at speeds of up to 5–10 km/h while covering depths from less than 1 m to several hundred meters [18,19]. Table 1 contrasts the key performance indicators of the main methods, demonstrating the engineering applicability and development potential of TEM for detecting underground spaces in urban areas.

However, when this exploration approach, originally designed for resource prospecting in open fields, is applied to modern cities, the operational environment changes drastically. Unlike electromagnetically pristine fields, urban landscapes are inundated with power-line electromagnetic grids composed of interwoven 220 V/50 Hz high-voltage lines [20]. Furthermore, dense clusters of buildings and heavy traffic severely restrict the space available to lay large transmitter loops. In such confined, high-interference environments, the ability of urban TEM technology to precisely distinguish weak transient responses from complex, overlapping signals has become a critical technical challenge that the industry must urgently overcome.

**Figure 1:**
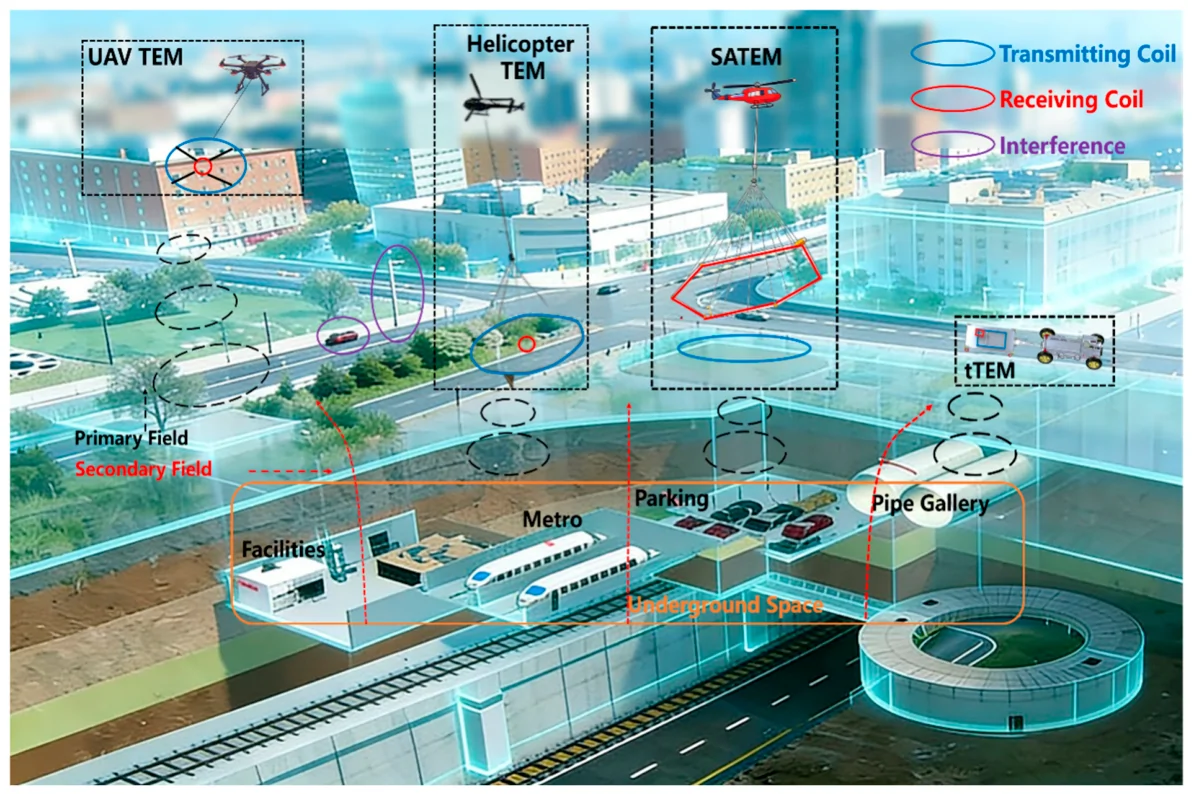
Conceptual schematic of four mobile transient electromagnetic (TEM) acquisition configurations for urban underground-space detection. The four platform types include UAV-borne TEM, helicopter-borne TEM, semi-airborne TEM, and ground-towed TEM. The diagram illustrates representative urban surface features and subsurface targets, including buildings, underground facilities, metro infrastructure, parking structures, and utility/pipeline galleries, together with the transmitter and receiver coils, primary and secondary electromagnetic fields, and representative interference sources. The illustration is conceptual and is not drawn to a quantitative spatial scale.

The core of this challenge is driving the evolution of sensing platforms. To cope with the complexity of urban environments, sensing platforms have evolved from mere mechanical supports for equipment into intelligent terminals that integrate high-frequency transmission, ultra-fast turn-off capabilities, and adaptive noise-reduction algorithms. This paradigm shift is reflected in the suppression of shallow blind zones via hardware metrics and in the transformation of detection modalities. Traditional ‘fixed-point static measurements’ are rapidly being replaced by ‘dynamic continuous scanning’ due to their low efficiency and insufficient lateral resolution. To accommodate this trend, mobile transient electromagnetic exploration technologies, such as towed TEM [21], semi-airborne TEM [22], helicopter-borne TEM [23], and UAV-borne TEM [24], have become increasingly popular (Figure 1). These motorized detection modalities imply that sensing platforms must possess exceptional mobility [25]. They utilize flexible carrier configurations to bypass ground obstacles and achieve non-contact, high-precision detection at construction sites, at demolition ruins, or in restricted-access areas.

This review aims to provide a systematic overview of sensing platform technologies for the transient electromagnetic method, as applied to the detection of underground urban spaces. Section 2 focuses on key technical aspects of urban TEM systems, including small-loop and high-frequency transmission technologies, anti-interference approaches, and imaging/inversion methodologies, with horizontal comparisons of representative techniques provided to clarify their advantages, limitations, and applicable conditions. Section 3 concentrates on the evolutionary trajectory, application progress, and comparative assessment of different mobile TEM platforms. It analyses the advantages and limitations of ground-towed platforms for improving survey efficiency and lateral resolution, and compares their applicability with that of airborne and semi-airborne configurations. It also clarifies the evolution of airborne sensing platforms from traditional manned systems to lightweight UAV-based platforms and ground–air cooperative semi-airborne modes. Building on this, Section 4 discusses the key engineering challenges and implementation bottlenecks of TEM sensing platforms in practical urban applications. Finally, Section 5 summarizes the conclusions of this review and discusses future directions for development and potential technological pathways for urban TEM detection. By comprehensively reviewing technological advances, engineering challenges, and future opportunities, this paper aims to provide insights and technical references for the development of fine-scale detection of underground urban spaces.

Several high-quality reviews of TEM technology have been published, but their scopes differ significantly from that of the present work. Lin et al. [23] reviewed the advances of helicopter time-domain electromagnetic technology in China, focusing on a single carrier category and mainly on mineral and groundwater applications; Wu et al. [26,27] summarized the development of helicopter-borne and semi-airborne systems, again from the perspective of one platform family; Parshin et al. [24] concentrated on lightweight UAV-TEM hardware; and Auken et al. [19] reviewed the towed tTEM platform mainly for hydrogeological mapping in open, low-noise farmland. None of these reviews systematically addresses the coupled constraints of urban scenarios—strong power-frequency interference, dense metallic infrastructure, confined operation space, and meter-scale engineering targets—nor do they treat the sensing platform as an integrated chain coupling transmission, noise suppression, imaging, and carrier. The present review differs from previous work in three aspects: (i) it is organized around the full urban sensing chain (small-loop transmission, anti-interference, imaging interpretation, and mobile carriers) rather than around individual instruments; (ii) it provides horizontal, quantitative comparisons of representative decoupling structures, noise-suppression algorithms, imaging strategies, and platform parameters under unified metrics (Table 2, Table 3, Table 4, Table 5, Table 6 and Table 7); and (iii) it explicitly evaluates the applicability, technological maturity, and unresolved limitations of each platform family in typical urban working conditions (Section 3.4), thereby offering direct guidance for engineering scheme design.

## 2. Key Technological Aspects

### 2.1. Small-Loop and High-Frequency Technology

In urban underground space exploration tasks, site-specific operational constraints make it difficult to deploy traditional large-loop systems. Consequently, the small-loop TEM has demonstrated unique value for applications due to its compact working footprint. However, this transition towards miniaturization is not simply a reduction in physical dimensions. Rather, the core technical challenge lies in balancing transmitter power, the turn-off blind zone, and signal fidelity within an extremely short sounding distance. Due to the severe self- and mutual inductance inherent in small-loop systems, secondary-field signals in the early time domain are highly susceptible to contamination from residual primary-field signals [28]. To address this issue, Xu et al. [29] proposed a dipole integral superposition method. By decomposing the transmitting source into an infinite number of electric dipoles, they resolved the computational oscillation issue associated with small loops in forward modeling for layered media. This established a theoretical benchmark for shallow, fine-scale exploration.

To suppress primary field interference at the hardware level and enhance the performance of shallow, fine-scale exploration, researchers have focused on developing structural decoupling techniques and optimizing turn-off circuits. Fu et al. [30] proposed a crossing-loop configuration that strongly suppresses mutual inductance by geometrically canceling magnetic flux. This improves the accuracy of identifying shallow, low-resistivity targets. Shi et al. [31] developed non-coplanar positive compensation decoupling technology by coaxially configuring the compensation loop with the transmitter loop, thereby enhancing structural stability and achieving high-precision removal of the early-time primary field. Furthermore, Chen et al. [32] investigated an opposing-coils differential measurement method that directly suppresses inductive coupling interference at the physical source, thereby enabling pure secondary-field observations. Hao et al. [33] observed that the TEM transmitter system is a core component of exploration equipment and directly affects detection accuracy and device stability. To this end, they developed a circuit that integrates impedance matching, preloading, and active constant-voltage clamping technologies. This effectively reduces overshoot on the rising edge and achieves rapid, linear turn-off on the falling edge. To address the prolonged turn-off time caused by inductive loads, Tan et al. [34] designed a second-order fast discharge circuit topology. This technology makes the turn-off time independent of the current magnitude within a certain range. It consistently maintains a 58 μs turn-off time at both 9 A and 50 A, thereby providing hardware support for high-power shallow detection. Building on this, Wang et al. [35] introduced passive clamping and oscillation absorption technologies. Using a bucking coil to reduce the field intensity at the center point, in conjunction with a dual-sampling-rate receiver, achieved high-precision splicing of responses from triangular and trapezoidal waves. For multi-turn small-loop systems, Yan et al. [36] optimized the H-bridge driving logic to design a high-frequency transmitter circuit, thus further validating the feasibility of such circuits in complex urban environments.

In terms of exploration strategies, a single waveform often struggles to meet the resolution requirements of different depths simultaneously. Liu et al. [37] proposed a multi-pulse combined transmission scheme that employs dual-pulse energy-enhancement technology to achieve synchronous coverage of both shallow and deep targets. Li et al. [38] introduced a composite excitation strategy combining trapezoidal and semi-sinusoidal waves, leveraging the latter’s high-frequency characteristics to extract fine details. In contrast, the flat-top segment of the trapezoidal wave ensures energy penetration into deeper structures [39]. In addition, to meet the high-frequency requirements of airborne or pod-based platforms, Liu et al. [40] developed a bipolar semi-sinusoidal inverter that utilizes the RLC series resonance principle to generate high-amplitude currents from low-voltage sources, significantly enhancing excitation efficiency.

Although various small-loop decoupling and fast turn-off technologies have achieved significant progress, their engineering performance differs considerably under urban operating conditions. Geometric decoupling approaches, including crossing-loop and compensation-loop configurations, directly suppress mutual inductive coupling at the hardware level and are effective for improving early-time signal quality. However, their performance is highly dependent on the stability of the coil geometry and on platform vibration. In contrast, circuit-based fast turn-off techniques improve current decay characteristics and reduce switching transients, thereby offering better adaptability for mobile platforms. Nevertheless, these approaches cannot eliminate residual primary-field contamination in the ultra-early-time window. Therefore, future urban TEM systems are likely to adopt hybrid architectures that combine weak-coupling sensor structures, optimized transmitter circuits, and advanced signal-processing algorithms, rather than relying on a single suppression mechanism.

**Figure 2:**
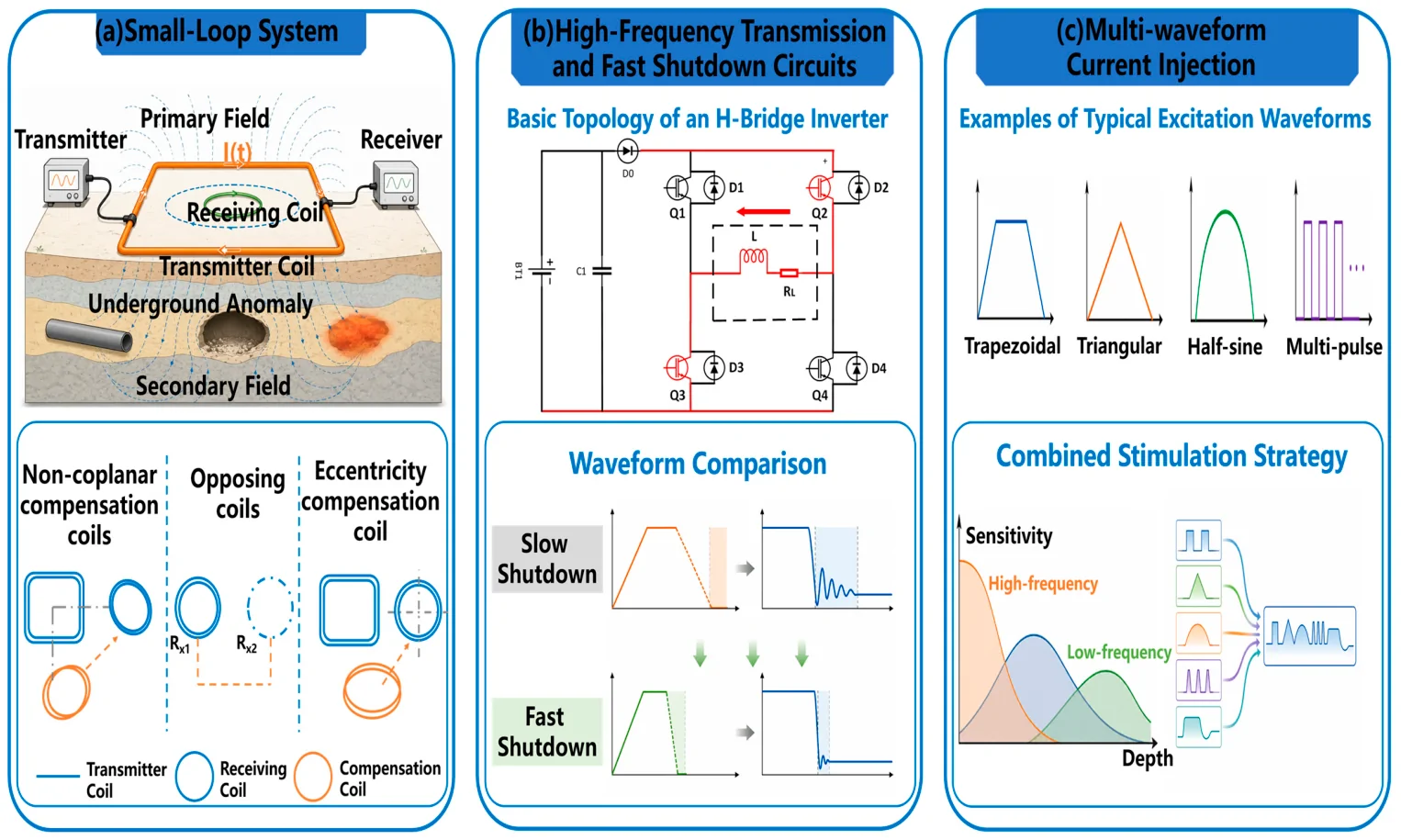
Schematic overview of small-loop and high-frequency transmission technologies for urban TEM systems. (**a**) Small-loop transmitter–receiver configurations and representative structural decoupling schemes, including non-coplanar compensation, opposing-coil, and eccentric-compensation configurations, which are used to reduce primary-field coupling and improve early-time secondary-field acquisition. (**b**) High-frequency transmission and fast turn-off circuitry, illustrating the H-bridge transmitter topology and the difference between relatively slow and fast current turn-off processes; a shorter turn-off time reduces the early-time blind zone. (**c**) Representative excitation waveforms and combined multi-waveform transmission strategies, including trapezoidal, triangular, half-sine, and multi-pulse excitation, which balance shallow-resolution requirements and energy delivery for deeper exploration. Tx and Rx denote transmitter and receiver, respectively.

In summary, small-loop and high-frequency transmission technologies entail more than just reducing single-device dimensions; they represent a systematic optimization process that includes weakly coupled loop structures, fast-turn-off circuits, and multi-waveform excitation strategies. Figure 2 visually encapsulates these technical pathways by summarizing the small-loop system configurations, fast-turn-off circuits, and multi-waveform transmission strategies. The future evolution of this technology will converge in two main areas: firstly, the synergistic design of weakly coupled loop structures, compensation coils, and fast turn-off circuits to further reduce the early-time blind zone and improve the quality of shallow effective signals; and secondly, multi-waveform combination excitation and digital waveform control to improve shallow resolution while ensuring sufficient energy for deep exploration. Synergistic optimization of hardware and software is key to enabling small-loop TEM to adapt to complex urban environments and achieve high-precision continuous detection.

To move beyond a simple catalog of individual achievements, Table 2 provides a horizontal comparison of the representative small-loop decoupling structures and fast turn-off circuits reviewed above, using turn-off behavior, blind-zone suppression mechanism, and engineering constraints as unified metrics. Three observations can be drawn. First, geometric decoupling structures (crossing-loop, non-coplanar bucking, and opposing-coils configurations) suppress the primary field physically at the source and therefore most directly compress the early-time blind zone. Still, their suppression performance degrades once the coil attitude or relative geometry is disturbed, which is almost unavoidable during mobile profiling on uneven urban roads. Second, circuit-level schemes (constant-voltage clamping, second-order fast discharge, and passive clamping with oscillation absorption) shorten and linearize the turn-off edge regardless of loop geometry; the second-order fast discharge topology, for example, keeps the turn-off time constant at 58 μs over a 9–50 A current range [34], which is critical for high-power shallow sounding. However, a residual microsecond-scale blind zone cannot be fully removed. Third, structural and circuit-level schemes are complementary rather than competitive: the best-performing urban systems (e.g., JLU-UtTEM, CUGTEM-TY, and FCTEM60 in Table 5) invariably combine weak-coupling loop structures with fast turn-off circuits and dual-sampling-rate receivers. It should be emphasized that all of these hardware measures can only suppress, rather than eliminate, the shallow blind zone; in the ultra-shallow range of 0–3 m, residual primary-field ringing and early-time noise still limit the reliable identification of small cavities, particularly where metallic pipelines overlap with the target response.

**Table 2:** Horizontal comparison of representative small-loop decoupling structures and fast turn-off circuits.

Scheme	Working Principle	Reported Performance	Shallow Blind-Zone Suppression	Main Limitations	Ref.
Crossing-loop configuration	Geometric cancelation of mutual magnetic flux between transmitter and receiver loops	Mutual inductance strongly suppressed at the source; improves identification of shallow low-resistivity targets.	High (physical suppression at source)	Requires precise geometric alignment; sensitive to coil attitude and terrain undulation	[30]
Non-coplanar bucking compensation	A compensation loop configured coaxially with the transmitter loop cancels the primary flux at the receiver position.	High-precision removal of the early-time primary field; good structural stability	High	Residual coupling reappears once the relative geometry changes during mobile survey.	[28,31]
Opposing-coils differential measurement (OCTEM)	Two oppositely wound coils measure differentially, canceling inductive coupling.	Enables near-pure secondary-field observation; supports ultra-shallow continuous profiling	High	Reduced effective transmitter moment; detection depth is traded for shallow resolution	[32,41]
Constant-voltage clamping with impedance matching	Active clamping and pre-loading shape the transmitter-current edge	Reduced rising-edge overshoot; rapid, quasi-linear falling edge	Medium (shortens the turn-off ramp)	Higher circuit complexity and power dissipation	[33]
Second-order fast discharge circuit	The second-order discharge topology decouples the turn-off time from the current amplitude.	Constant 58 μs turn-off time maintained at 9–50 A	Medium-high	Residual μs-scale blind zone remains; thermal stress at high current	[34]
Passive clamping + oscillation absorption + dual sampling rate	Bucking coil lowers the central-point field; dual-rate receiver splices early and late data	High-precision splicing of triangular/trapezoidal wave responses	Medium-high	Calibration burden of dual-rate splicing	[35]
High-frequency multi-turn small-loop transmitter	Optimized H-bridge driving logic for high transmission frequency	Feasible high-frequency excitation for multi-turn small loops	Medium	Increased mutual inductance of multi-turn loops must be compensated	[36]

Notes: The qualitative blind-zone-suppression levels (High, Medium-high, and Medium) are comparative assessments synthesized from the performance and limitations reported in Refs. [28,30,31,32,33,34,35,36,41] rather than values obtained from a unified experimental benchmark. Reported performance corresponds to the individual laboratory or field conditions of the cited studies.

### 2.2. Noise Suppression

**Figure 3:**
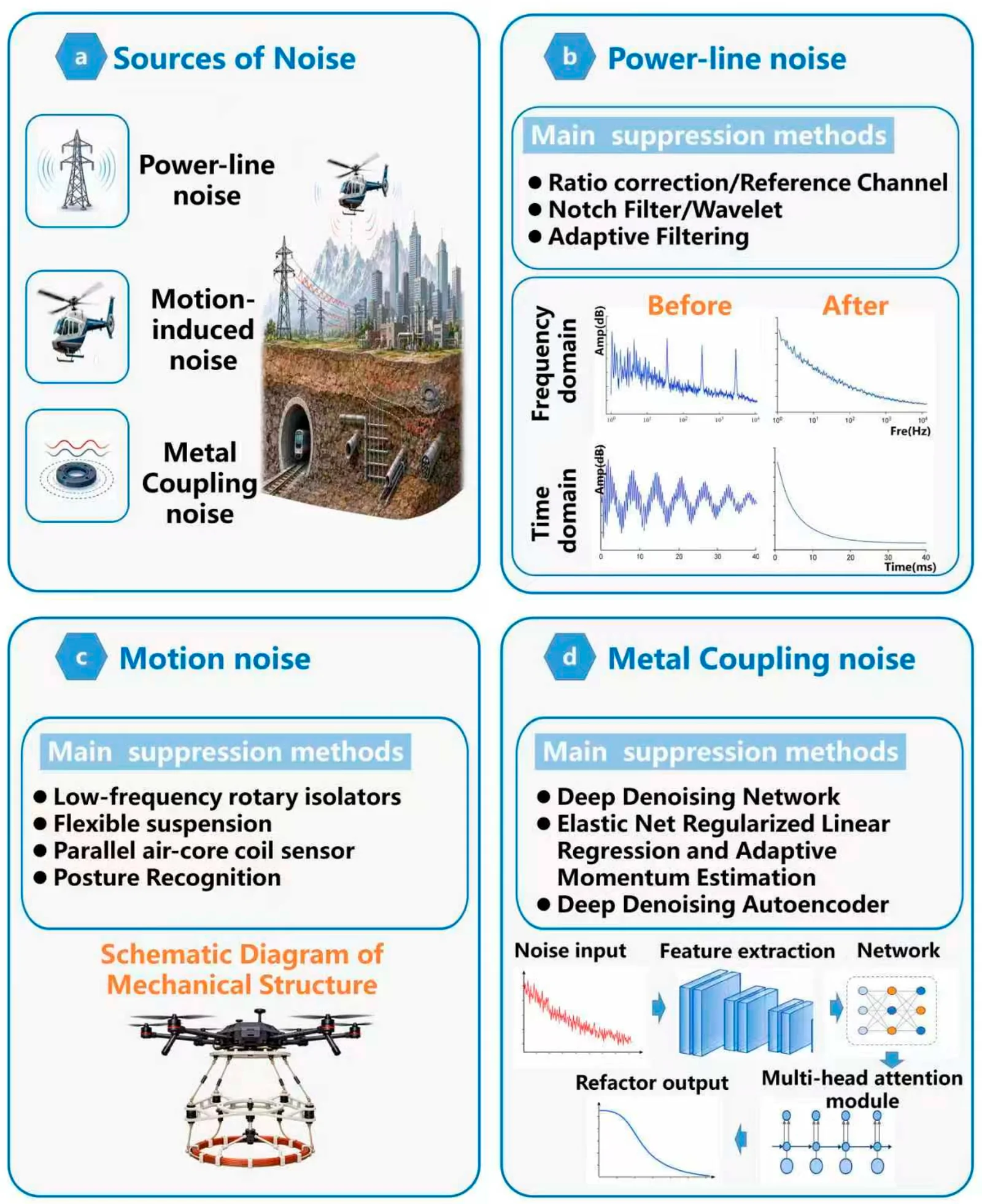
Schematic overview of major noise sources and corresponding suppression strategies in urban TEM measurements. (**a**) Representative urban interference sources, including power-line interference, motion-induced noise (MIN), and electromagnetic coupling from metallic infrastructure. (**b**) Suppression of power-line interference using reference-channel/ratio correction, notch or wavelet-based processing, and adaptive filtering; the frequency- and time-domain sketches illustrate the signal characteristics before and after suppression. (**c**) Mitigation of motion-induced noise through low-frequency rotational isolation, flexible suspension, parallel air-core coil sensors, and platform-attitude monitoring. (**d**) Suppression and reconstruction of metallic-coupling interference using regression-based methods, adaptive optimization, and deep-learning denoising networks. MIN denotes motion-induced noise.

TEM exploration in urban environments is confronted with extremely complex interference sources, including power-line noise, motion-induced noise (MIN) from vehicles, and robust electromagnetic coupling effects from subsurface metallic pipelines and maintenance hole covers. Through forward modeling, Liu et al. [42] demonstrated that reducing MIN levels significantly increases the system’s detection depth. Furthermore, An et al. [43] found that low-resistivity pipelines shield the electromagnetic field, creating a weak field zone directly beneath them and distorting the boundaries of anomalous bodies in inversion imaging. Consequently, developing multidimensional anti-interference technologies is essential for achieving high-precision urban TEM exploration. To address these issues, existing anti-interference methodologies advance primarily along three pathways: adaptive time-frequency suppression of power-line noise; physical isolation of motion-induced noise; and data-driven, multi-dimensional disturbance rejection coupled with algorithmic optimization. The fundamental logic of urban TEM anti-interference techniques is summarized in Figure 3.

Power-line interference and its harmonics are the most prevalent form of environmental noise in urban TEM. They are characterized by frequency drift and envelope modulation, which render traditional fixed-parameter notch filters ineffective. Initial attempts employed digital notch filtering and least-mean-squares (LMS) adaptive filtering; however, the extremely narrow stopbands often caused the Gibbs phenomenon, resulting in severe distortion of early-time signals [44,45]. Due to significant spectral overlap between power-line noise and genuine signals, researchers have proposed strategies for reconstructing interference using mathematical models. These strategies achieve physical cancelation by parameterizing and reconstructing the fundamental frequency, amplitude, and phase of the harmonics [46]. The remote reference (RR) technique uses noise mapping between the main receiver loop and a reference coil to establish relationships during signal-free time segments, thereby isolating co-frequency interference driven by spatial correlation [47,48]. To address frequency non-stationarity, Huang et al. [49] proposed an enhanced Wavelet-NGSE algorithm to dynamically track subtle frequency shifts. Meanwhile, Sun et al. [50] introduced a genetic algorithm to identify optimal noise segments through feature matching for subtraction. Contemporary research has evolved into a combined strategy of mathematical modeling and intelligent optimization, achieving dynamic locking and deep stripping of time-varying power-line interference [51].

In rigidly connected airborne TEM systems, the angular vibration of the receiver coil within the geomagnetic field generates motion-induced noise (MIN), which often masks weak geological signals. Because its frequency often overlaps with the transmitter’s, conventional filtering is ineffective at eliminating it. To suppress MIN at its source, researchers have developed various mechanical isolation methods. For example, Liu et al. [42] designed a cable-parallel air-core coil sensor (CPACS) that suspends the receiver coil with elastic ropes. This suppresses the coil’s mechanical fundamental frequency well below the transmitter frequency, significantly reducing measured MIN levels. Sunderland et al. [52] developed a rotational vibration isolator based on the principle of neutral buoyancy. Using a fluid between concentric spherical shells and ultra-soft springs yields an exceptionally low resonant frequency, resulting in rotational vibration transmissibility far below that of commercial systems at low frequencies. The mechanically resonant coil sensor designed by Liu et al. [53] shifts the characteristic frequency of motion-induced noise below the transmitter frequency by modifying the coil support structure, successfully achieving frequency-domain separation of signal and noise. These physical isolation methods bypass post-processing algorithms, neutralizing motion-induced noise contamination directly at the source.

For the non-stationary electromagnetic noise and intense coupling interference from metallic facilities in urban areas, data-driven approaches have been shown to perform better. The BA-ATEMNet deep denoising network, proposed by Wang et al. [54], effectively extracts transient responses from intense power-line noise backgrounds by integrating Bayesian learning with a multi-head self-attention mechanism. To address primary multiplicative noise (PMN) caused by bird-swing in helicopter-borne TEM systems, Xu et al. adopted elastic-net regularized linear regression and the adaptive moment estimation (Adam) algorithm [55]. This substantially advanced the earliest usable time of the data and significantly enhanced shallow-detection capability. Zhang et al. [56] further streamlined this methodology by rapidly suppressing early-time signal noise using regression coefficient vectors derived solely from induced-voltage data, thereby demonstrating exceptional processing efficiency. To mitigate localized, intense interference from metallic maintenance hole covers and passing vehicles on urban roads, An et al. [57] developed a pipeline that combines a deep denoising autoencoder with iteratively reweighted least squares (DDAE-IRLS). This automatically identifies contaminated data segments and performs high-fidelity reconstruction, enabling rapid surveys of road hazards. Furthermore, Ran et al. [58] reduced the impact of environmental coupling noise at the source by optimizing the positions of the transmitter and receiver loops to modify the electromagnetic coupling path.

The paradigm of urban TEM anti-interference technology is shifting from passive noise reduction in the post-processing phase to active disturbance rejection throughout the entire exploration workflow. At the physical sensing layer, motion-induced noise and geometric coupling errors are mitigated directly at the source by mechanical vibration isolation, flexible suspension, bucking coils, and attitude sensing. At the data processing layer, adaptive filtering, reference channel calibration, deep denoising networks, and regression reconstruction further isolate power line noise, metallic facility responses, and true geological anomalies. Future anti-interference technologies should focus on integrating sensor structures, auxiliary observational data, and intelligent algorithms synergistically to improve the interpretability and reliability of weak TEM responses in complex urban environments.

A closer look at data-driven approaches reveals trade-offs that are rarely discussed explicitly. BA-ATEMNet [54], which couples Bayesian learning with a multi-head self-attention mechanism, achieves strong suppression of intense power-line noise and provides uncertainty estimates, but its reconstruction fidelity for the weakest early-time secondary-field samples depends heavily on the similarity between the training set and the local noise background, and its network size makes onboard real-time deployment demanding. The DDAE-IRLS pipeline [57] automatically localizes contaminated segments caused by metallic covers and passing vehicles and reconstructs them with iteratively reweighted least squares; because only contaminated segments are reconstructed, the fidelity of uncontaminated early-time signals is well preserved, and the computational cost is lower than that of end-to-end networks, making it suitable for near-real-time processing of towed road surveys. By contrast, the regression-based suppression of primary multiplicative noise [55,56] relies on the correlation between bird swing and the induced noise rather than on training data and is therefore transferable between survey areas; however, its linearity assumption breaks down under strongly nonlinear motion. Recent expert-system and hybrid deep models—such as the deep learning expert system for automated TEM processing [59] and the PC-BiLSTMNet hybrid denoiser [60]—further improve generalization by embedding physical constraints into the network, at the price of larger training-data requirements. In summary, model-driven methods remain preferable when training data are scarce or real-time processing is mandatory. In contrast, deep networks outperform them under intense, non-stationary urban noise provided that representative training sets are available (Table 3).

**Table 3:** Comparison of representative noise-suppression methods for urban TEM under unified metrics.

Method	Target Noise	Early-Time Signal Fidelity	Computational Cost/Real-Time Capability	Dependence on Auxiliary Data or Training	Ref.
Fixed notch/LMS adaptive filtering	Stationary power-line harmonics	Low (Gibbs distortion of early-time gates)	Low cost; real-time	None	[44,45]
Model-based harmonic reconstruction	Power-line fundamental and harmonics	Medium-high (physical cancelation without filtering)	Medium; near real-time	Requires accurate noise-parameter estimation	[46]
Remote reference (RR)	Spatially correlated power-line noise	Medium-high	Medium	Requires a reference coil and signal-free segments	[47,48]
Wavelet-NGSE	Power-line noise with frequency drift	Medium	Medium	None	[49]
Genetic-algorithm noise-segment matching	Non-stationary power-line noise	Medium	Medium-high	Requires identifiable noise segments	[50]
Mechanical isolation/resonant coil (CPACS, rotational isolator)	Motion-induced noise	High (noise removed at source, no signal distortion)	No computation; hardware only	None	[42,52,53]
Regression-based PMN suppression (elastic-net Adam; regression coefficients)	Primary multiplicative noise from bird swing	Medium-high (advances the earliest usable time)	Low; suitable for onboard processing	None (no training data)	[55,56]
Deep denoising networks (BA-ATEMNet, DDAE-IRLS, expert system, PC-BiLSTMNet)	Intense power-line noise; localized metallic interference	Potentially high, but weak early-time signals risk over-smoothing if the training set is unrepresentative	High training cost; inference feasible on edge devices after compression	Heavy reliance on representative training datasets	[54,57,59,60]
KPCA-based denoising	Atmospheric noise; power-line noise; coupling noise	Medium-high (nonlinear feature extraction and signal reconstruction)	Medium (statistical analysis-based processing)	None (data-driven without training samples)	[61]
Variational Bayesian adaptive Kalman filtering (VBAKF) method	Motion-induced noise; Power-line noise;	High (adaptive state estimation and signal preservation)	Medium (recursive filtering with online covariance estimation)	Requires exponential decay model assumption	[62]
Recurrent self-coding neural network (RSCN)	Motion-induced noise; atmospheric noise; anthropogenic noise	High (weak-signal recovery and signal-feature preservation)	Medium-high; efficient full-line denoising	Requires representative training datasets	[63]

Notes: Fidelity and cost are qualitatively ranked based on the evidence reported in the cited studies; a unified public benchmark for urban TEM denoising remains lacking.

In complex noise environments for transient electromagnetic (TEM) exploration, including power-frequency interference, motion-induced noise (MIN), atmospheric noise, and metallic infrastructure coupling interference, existing studies have gradually evolved from single-source noise suppression to multi-type noise collaborative mitigation. Chen et al. [61] employed kernel principal component analysis (KPCA) to extract nonlinear features from TEM decay curves, enabling simultaneous suppression of atmospheric noise, power-line interference, and anthropogenic infrastructure coupling noise without requiring training datasets. However, its adaptability to different survey areas remains affected by variations in statistical characteristics of the acquired data. Wu et al. [62] proposed a variational Bayesian adaptive Kalman filtering (VBAKF) method that jointly suppresses motion-induced and power-line noise by online estimation of the noise covariance. This approach provides favorable real-time processing capabilities, although its performance relies on the assumption of an exponential signal decay model. Lin et al. [63] developed a recurrent self-coding neural network (RSCN) by integrating recurrent neural networks, convolutional encoding, and attention mechanisms, thereby achieving the integrated removal of motion-induced, atmospheric, and anthropogenic noise. The proposed method demonstrates improved weak-signal recovery capability and long-profile real-time processing performance; however, it still requires sufficient training samples to ensure model reliability. Overall, multi-type noise collaborative suppression technologies have evolved from conventional statistical analysis methods to hybrid approaches that combine model-driven strategies and deep learning techniques. Although significant improvements have been achieved in denoising accuracy, real-time capability, and weak-signal preservation under complex noise conditions, deep learning-based methods remain highly dependent on training datasets and model generalization. At the same time, traditional statistical and model-driven approaches require further improvement in adaptability to complex non-stationary noise environments.

### 2.3. Imaging Technology

**Figure 4:**
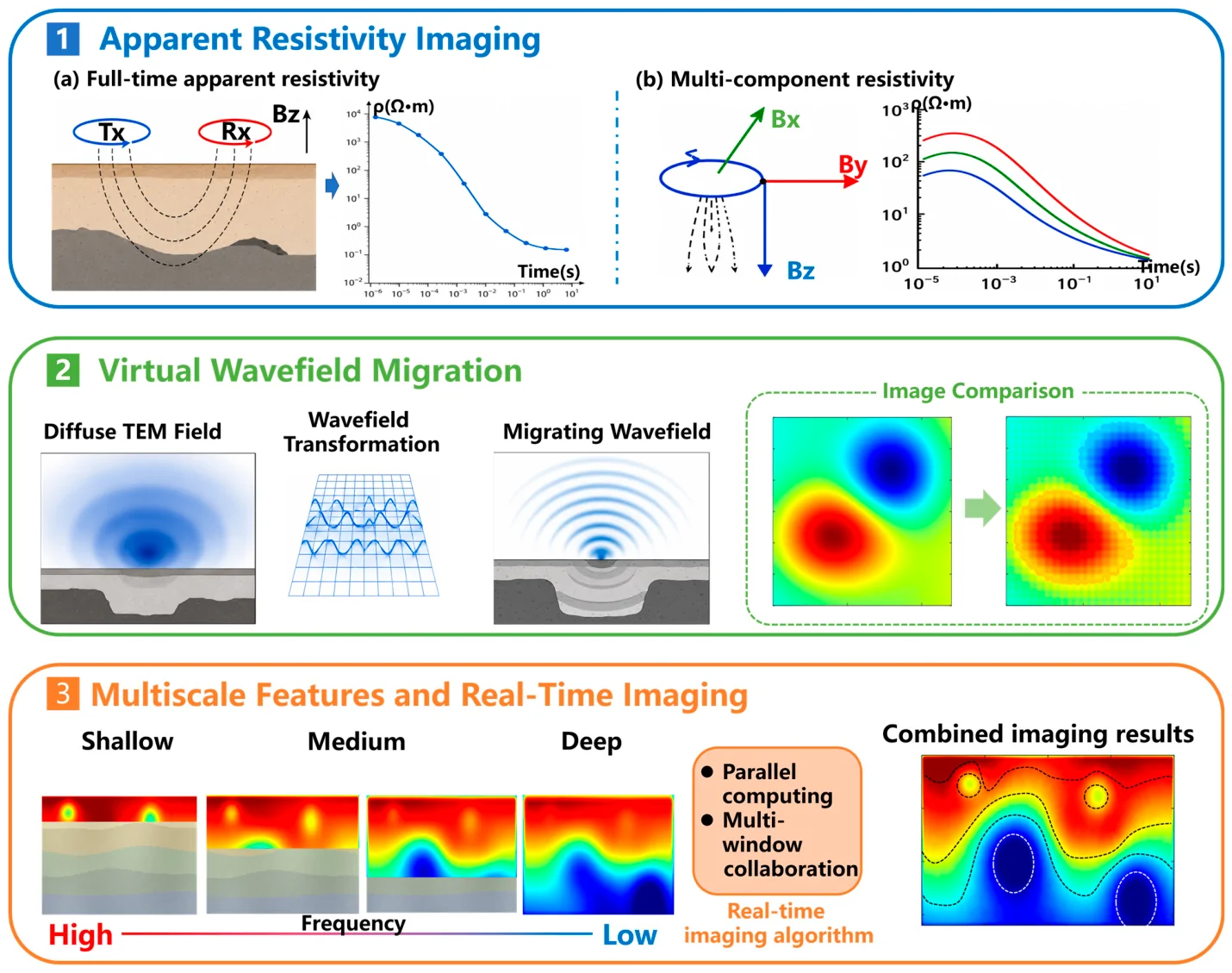
Schematic overview of representative TEM imaging technologies for urban underground-space detection. The framework summarizes three complementary imaging pathways: apparent-resistivity imaging based on single- or multi-component electromagnetic responses; virtual-wavefield migration (VWM), in which the diffusive electromagnetic response is transformed into a virtual wavefield to enhance structural boundaries; and rapid/multi-resolution imaging and inversion, which combine data at different spatial or temporal scales to balance shallow-detail recovery, deep-structure reconstruction, and computational efficiency. The input TEM decay responses are converted into resistivity or structural images that support the identification and delineation of subsurface anomalies. VWM denotes virtual-wavefield migration.

The efficiency and precision of TEM imaging technologies are key to realizing the vision of transparent cities when exploring urban underground spaces. Traditional inversion methodologies involve significant computational demands, making them unsuitable for rapid surveys. Consequently, investigating novel, high-resolution, high-efficiency detection and imaging approaches has become a critical issue that must be urgently addressed [64]. In recent years, the research paradigm has shifted from simplistic single-parameter deduction to the precise refinement of apparent resistivity definitions, microscale virtual-wavefield transformation imaging, and multi-resolution, real-time imaging strategies tailored to complex urban environments. Recent studies have further extended TEM inversion from resistivity reconstruction toward quantitative geological interpretation by integrating electromagnetic responses with petrophysical parameters [65]. The overarching research framework is outlined in Figure 4.

As the most intuitive interpretive parameter, the accuracy of the apparent resistivity definition directly affects the quality of imaging outcomes. Zhu et al. [66] noted that traditional apparent resistivity definitions consider only the vertical magnetic field component and rely on the monotonic relationship between resistivity and electromagnetic responses. To expand the dimensionality of exploration, researchers have begun investigating multi-component imaging. For example, Cheng et al. [67] proposed a methodology for defining apparent resistivity based on the step response of the electric field component. This approach leverages electric field data to enhance the resolution capability for deep-seated structures. A similar paradigm shift has occurred at the signal-source level: Fan et al. [68] incorporated differential-pulse waveforms into ground-to-air TEM apparent-resistivity imaging. This introduced an all-field apparent resistivity definition based on differential pulses, thereby enhancing imaging resolution.

The evolution of apparent resistivity definitions optimizes the ‘interpretive toolkit’. At the same time, the emergence of virtual wavefield migration (VWM) technology fundamentally rewrites the inherent limitations of the TEM diffusion equation, namely its low resolution and severe diffusion-induced smoothing effects, from the mathematical bedrock [69]. Qi et al. [70] observed that diffuse electromagnetic fields have a distinctive mathematical integral representation that can be transformed into a virtual wavefield that satisfies the wave equation. This wavefield transformation enables mature migration imaging methods used in seismic interpretation to be applied to the TEM domain. In their monograph, Li et al. [71] systematically expounded on migration imaging theory and demonstrated, using field datasets, that it can clearly delineate stratigraphic horizons. The interpreted results aligned exceptionally well with actual geological conditions. Building on this, Li et al. [64] advanced a multi-resolution virtual wavefield migration imaging approach. By performing multi-scale fusion of virtual wavefields across different frequencies, they successfully reconciled the issue of a single frequency struggling to balance shallow details and deep structural frameworks simultaneously.

In practical urban operational scenarios, subsurface targets usually exhibit clear multiscale features, ranging from small-scale localized anomalies, such as shallow pipelines and cavities, to large-scale continuous features, such as deep aquifers, fault zones, and major underground infrastructure. To address this challenge, Li et al. [72] successfully extracted response information across a range of frequencies and scales by employing multi-source excitation, differential-pulse scanning, and multi-window virtual wavefield transformations. Meanwhile, real-time imaging has become a mandatory requirement for rapid urban surveys. The generalized 1-D rapid imaging method proposed by Christensen [73] can adapt to various measurement configurations, transmitter waveforms, and field components. Furthermore, Li et al. [74] used extreme learning machines (ELMs) to rapidly map TEM responses to resistivity configurations. This significantly reduced the computational overhead of conventional iterative inversions and provided crucial technical support for the near-real-time processing of large-scale urban scanning data.

Contemporary advancements in imaging technologies are progressively shifting from single apparent resistivity mapping towards a balanced approach that emphasizes multi-component parameterization, wave-field transformation, and rapid, real-time imaging. Multi-component apparent resistivity definitions increase sensitivity towards complex electrical structures, while virtual wavefield migration techniques mitigate the blurring effects inherent in diffusion field imaging. Furthermore, multi-scale rapid imaging methods provide robust support for the near-real-time processing of large-area, continuous urban-scanning data. The profound integration of these technologies will make “scan-and-see” dynamic imaging an essential foundation for the fine-scale management of future urban underground spaces.

The conditions applicable to the different apparent resistivity definitions warrant explicit clarification. Single-component (vertical magnetic field) definitions [66] assume a monotonic relationship between response and resistivity. They are adequate for quasi-layered media, but they lose monotonicity and, therefore, uniqueness over complex three-dimensional urban targets. The electric-field step-response definition [67] is more sensitive to deep, relatively resistive structures. It is better suited to electric-source or grounded-source configurations. In contrast, the differential-pulse full-domain apparent resistivity [68] removes the waveform dependence of conventional definitions and is particularly suitable for ground-air (semi-airborne) configurations with non-standard excitation. In terms of anomaly types, vertical-component apparent resistivity responds most strongly to water-bearing cavities and conductive clay interlayers; multi-component and electric-field definitions better constrain the boundaries of relatively resistive targets such as dry cavities and engineered concrete structures; and for highly conductive metallic pipelines, none of the apparent resistivity definitions alone can separate the pipeline response from the host medium-explicit decoupling or joint inversion incorporating pipeline parameters is required instead [41,56].

Table 4 compares the reviewed imaging strategies with conventional inversion in terms of computation time, characterization of shallow details, deep structural reconstruction, and robustness to strong urban noise. Conventional 1-D iterative inversion [6,29] provides quantitative layered models but requires minutes to hours per survey line and smooths shallow detail; 2-D smooth-constrained inversion [5] reconstructs deep structures more realistically at the cost of even heavier computation and a strong dependence on the initial model. Virtual wave-field migration [69,70,71] borrows seismic migration to sharpen diffusion-blurred boundaries and markedly improves the delineation of interfaces and cavity edges. Still, its wave-field transformation is noise-sensitive: under strong urban noise, the transformation integral amplifies high-frequency noise, so migration generally requires careful preceding denoising (Section 2.2), and its stability on raw urban data remains a recognized weakness. Multi-resolution virtual wave-field migration [64,72] alleviates the shallow-versus-deep trade-off at the cost of a multi-fold increase in computation. The generalized 1-D imaging of Christensen [73] and ELM-based rapid inversion [74] reduce processing latency to sub-second or near-real-time levels and are thus most compatible with towed and UAV-borne continuous profiling. However, their models are smoother and less resolved than iterative results. Physics-guided deep learning inversion [75] is emerging as a middle ground that balances near-real-time speed with improved structural fidelity. Finally, because vehicle-mounted and UAV-borne platforms impose strict payload and power budgets, real-time imaging on these platforms must also match algorithmic complexity to edge computing hardware (e.g., embedded GPUs or FPGAs). This engineering aspect has so far received little attention and warrants dedicated study.

**Table 4:** Comparison of TEM imaging and inversion strategies under urban operating conditions.

Method	Category	Computation Time (Typical)	Shallow Detail	Deep Reconstruction	Robustness to Strong Urban Noise	Ref.
Apparent resistivity mapping (single-/multi-component)	Qualitative to semi-quantitative imaging	Real-time	Medium (parameter-dependent)	Low-medium	Medium (robust but blurred)	[66,67,68]
Generalized 1-D imaging	Approximate quantitative imaging	Sub-second per sounding	Medium	Medium	Medium	[74]
ELM rapid inversion	Learning-based fast inversion	Near real-time	Medium (smoother models)	Medium	Medium-high once trained	[73]
Physics-guided deep-learning inversion	Learning-based quantitative inversion	Near real-time after training	Medium-high	Medium-high	High (noise-decoupled representation)	[75]
Virtual wave-field migration (VWM)	Wave-field transformation imaging	Seconds to minutes per profile	High (sharpened boundaries)	Medium	Low-medium (noise-sensitive transformation)	[69,70,71]
Multi-resolution VWM	Multi-scale wave-field imaging	Several times that of single-resolution VWM	High	Medium-high	Low-medium	[64,72]
Conventional 1-D iterative/2-D smooth inversion	Quantitative model inversion	Minutes to hours per profile	Medium (smoothed by regularization)	High (with adequate depth of investigation)	Medium-high (regularization suppresses noise)	[5,6,29]

Notes: Computation times are indicative values under typical workstation or edge-computing conditions as reported in the cited studies; actual performance depends on data volume, model dimension, and hardware. The qualitative ratings for shallow detail, deep reconstruction, and noise robustness are relative comparisons synthesized from the cited studies rather than results of a unified benchmark.

## 3. Sensing Platform in Urban Scenarios

The evolution of urban TEM sensing platforms can be divided into four stages, each driven by a distinct technical demand. In the first stage (before the 1990s), fixed-point static measurement with large loops dominated; systems were optimized for mineral prospecting in open fields, and their low efficiency and poor lateral resolution were acceptable because survey speed was not critical. In the second stage (1990s–2010s), the demand for rapid near-surface investigation drove loop miniaturization and the birth of ground mobile detection: the VETEM prototype (1994) [76] first demonstrated vehicle-towed ultra-shallow TEM, followed by the commercial AgTEM (2012) [18] and the tTEM system (2018) [19], which established continuous towed profiling as a mature modality. In the third stage (2000s–2010s), the need to bypass surface obstacles and restricted areas propelled airborne systems: manned helicopter platforms such as SkyTEM [77], AeroTEM [78], and the Chinese CHTEM series [23,79] matured rapidly, while their heavy magnetic moments and long turn-off times exposed a growing mismatch with shallow urban targets, directly motivating platform lightweighting. In the fourth, ongoing stage (late 2010s-present), lightweight UAV-borne systems [24,80,81] and ground-air cooperative semi-airborne configurations [27,82,83,84] have emerged to reconcile deep sounding with the constraints of confined urban airspace and payload limits, and the research frontier is shifting toward multi-platform cooperation and swarm sensing. The following subsections provide detailed reviews of the representative platforms for each family.

### 3.1. Ground Mobile/Towed Platforms

Traditional fixed-point TEM exploration faces challenges such as low operational efficiency, traffic disruption, and inadequate lateral resolution in complex urban environments. This makes it difficult to meet the requirements of rapid urban underground space surveys. To address these challenges, there has been a shift in ground-sensing platforms from static spot measurements to dynamic, continuous scanning. Through innovations such as small-loop system configurations, high-frequency, fast-turn-off technologies, and broadband, low-noise receiver techniques, researchers worldwide have developed multiple mobile/towed ground exploration systems tailored for urban scenarios. These systems effectively reconcile the contradiction between survey efficiency and detection precision. These systems’ core technical pathway relies on securing the relative positions of the transmitter and receiver loops with rigid frames to minimize motion-induced noise (MIN) from hardware vibrations, while incorporating bucking coils to mitigate primary-field interference and reduce shallow-sounding blind zones.

To address the contradiction between the limited penetration of ground-penetrating radar (GPR) in high-conductivity media and the insufficient shallow resolution of conventional TEM, the United States Geological Survey (USGS) and the University of Illinois Urbana-Champaign jointly developed the Very Early Time Electromagnetic System (VETEM) in 1994. Towed by an all-terrain vehicle (ATV), this platform was specifically engineered to resolve ultra-shallow imaging challenges. The core of the VETEM system resides in its flexible loop configurations, which primarily encompass two paradigms: (1) Separated Loop Configuration: The transmitter loop is placed horizontally to generate a vertical magnetic field, while the receiver consists of a set of orthogonal coils that independently perceive different magnetic field components, thereby facilitating the resolution of complex subsurface structures. (2) Eccentric Configuration: By finely adjusting the center-to-center offset between the transmitter and receiver loops, the primary field effect is neutralized via geometric relationships, significantly enhancing the signal-to-noise ratio (SNR) of weak early-time secondary fields [76]. In 2012, Groundwater Imaging Ltd. in Australia developed the AgTEM system. By introducing a bucking compensation coil between the transmitter and receiver loops, a coincident-loop, near-zero-coupling configuration was achieved via magnetic flux cancelation. This design effectively minimized the system footprint and enhanced topographic adaptability. The AgTEM system can operate at survey speeds up to 10 km/h, delivering a typical detection depth of 30 m (maximum 60 m) with a lateral resolution of approximately 10 m [18]. In 2018, the HydroGeophysics Group at Aarhus University in Denmark developed the tTEM system. This system comprises an ATV tow vehicle, a 2 m × 4 m single-turn transmitter loop, and a Z-component receiver coil. To circumvent the primary field turn-off delay that contaminates shallow signals in small-loop systems, a large-offset separated configuration was adopted, extending the transmitter-receiver separation to 9 m and the total system length beyond 14 m. To reconcile both shallow and deep detection requirements, the system features dual-moment excitation: a low-moment (LM) mode with a 2.8 A current and an ultra-fast turn-off time of 2.6 μs to minimize the shallow blind zone, and a high-moment (HM) mode with currents up to 30 A to guarantee deep-seated SNR. The tTEM system has been extensively deployed for geological mapping in Denmark, the United States, and Sweden [19].

Lin’s team at Jilin University developed the JLU-UtTEM exploration system. This platform integrates a transmitter loop and a receiver with a non-coplanar bucking compensation structure onto a non-metallic trailer chassis. The system is towed by an operational vehicle to enable continuous dynamic scanning. Using real-time dynamic sampling technology, the system provides uninterrupted coverage of subsurface strata while maintaining urban traffic flow. To adapt to urban paved roads, the team introduced a further innovation: a vehicular TEM detection device integrated into an electric car. Leveraging synchronous wheel-triggered technology, this system achieves real-time data transmission and concurrent imaging. It has been successfully applied to investigations of the weathering zone of bedrock roadbeds and assessments of urban groundwater hazards [57].

Meanwhile, Fu’s team at Chongqing University developed the FCTEM60 towed TEM system. This system integrates constant-voltage clamping for high-speed linear turn-off and crossing-loop decoupling to suppress the primary field. It also uses transient process correction technologies. The transmitter can deliver currents ranging from 60 to 120 A with a turn-off time of less than 85 μs. It is primarily used to investigate groundwater and geological hazards, predict tunnel and mine conditions, and detect grounding grids and pipelines. It plays a pivotal role in reducing the blind zone of electromagnetic sounding. Furthermore, the China University of Geosciences (Wuhan) and Wuhan Huarui have developed the CUGTEM-TY towed TEM system, which is based on the principles of the coplanar opposing-coils transient electromagnetic method (OCTEM). Through continuous measurement, it substantially suppresses the shallow ‘blind zone’ inherent in conventional TEM measurements. The system can perform ultra-shallow continuous profiling and deploy large central-loop arrays for deep-seated sounding, providing an efficient tool for rapidly inspecting roads and embankments. Table 5 summarizes the key technical parameters of the representative ground-mobile and towed TEM platforms discussed above. Unless otherwise stated, these values are nominal figures reported by the developers under favorable low-noise conditions, and typical and maximum values are distinguished where reported.

**Table 5:** Comparison of key technical parameters of mainstream ground mobile TEM sensing platforms.

Name	Developer/Source	Loop Configuration	Tx Current (A)	Turn-Off Time (μs)	Sampling Rate	Detection Depth (m)
VETEM	USGS, Denver, CO, USA	Separated/Eccentric	Small current	<1	GHz-level	<5
tTEM	Aarhus University, Aarhus, Denmark	Large-offset separated	2.8/30	2.6	250 kHz	0–70
AgTEM	Groundwater Imaging, Dubbo, NSW, Australia	Coincident, bucking-compensated (near-zero coupling)	12–50	16	-	1–100
JLU-UtTEM	Jilin University, Changchun, Jilin, China	Non-coplanar bucking	5–20	2.0–8.0	2 MHz/500 kHz	0–50
rTEM	Jilin University, Changchun, Jilin, China	Central loop	10	-	0.33 m interval trigger	0–20
CUGTEM-TY	CUG (Wuhan), Wuhan, Hubei, China	Coplanar opposing-coils	10	8	1 MHz/250 kHz	0–30
FCTEM60	Chongqing University, Chongqing, China	Crossing-loop decoupling	60–120	<85	1.25/2.5 MHz	Up to 200–300

Notes: The listed parameters are nominal values reported by the developers or in the cited literature under their respective test conditions; where both typical and maximum figures exist (e.g., AgTEM: 30 m typical, 60 m maximum detection depth), the maximum was obtained under favorable, low-noise conditions. Turn-off time refers to the transmitter-current fall time; sampling rate refers to the receiver A/D rate; ‘-’ denotes values not explicitly reported.

The future evolution of ground sensing platforms will focus on two key areas: first, utilizing receiver arrays to enhance multi-target feature extraction capabilities, and second, further compressing the sounding blind zone caused by turn-off delays through hardware optimization. Meanwhile, multi-physics integration (e.g., integrating electromagnetic and seismic wave sensors on a single platform) shows great potential for high-precision, fine-scale subsurface mapping in complex urban environments.

### 3.2. Airborne Platforms

In complex scenarios such as construction sites, demolition sites, large landfills, and locations with restricted access, traditional ground exploration methods often face multiple challenges, including limited operational space, low efficiency, and potential safety hazards. In contrast, airborne electromagnetic exploration platforms have natural advantages due to their non-contact operations. They can bypass the constraints imposed by ground obstacles, complex surface conditions, and restrictions on personnel access to sensor deployment sites. Despite stringent airspace controls in modern urban areas, the rapid, high-density ‘carpet’ scanning of large-scale construction zones or disaster sites from low altitudes using airborne platforms has become a key method of acquiring subsurface geoelectrical information in both shallow and deep layers.

Deploying airborne platforms for fine-scale urban surveys entails trade-offs among resolution, blind zones, and adaptability to complex electromagnetic environments. Early airborne sensing schemes relied primarily on manned helicopter-borne systems such as the SkyTEM system discussed by Auken [77] and the AeroTEM system by Balch [78]. Originally designed for prospecting at great depths, these systems ensured a strong signal by generating magnetic moments exceeding 105 A·m^2^. However, this ‘heavy-duty’ design paradigm has significant limitations in fine-scale engineering investigations. The large pod frames are highly susceptible to wind disturbances during flight, which induce intense bird-swing noise. Furthermore, prohibitive operational costs and strict requirements for take-off and landing sites severely limit their adoption in engineering surveys. Crucially, the turn-off times of manned platforms are frequently in the order of hundreds of microseconds, creating substantial early-time signal blind zones and rendering them incapable of imaging the shallow subsurface of urban underground spaces. The GEM-2A, developed by Won [85], was an early attempt to reduce the platform’s weight and enable multi-frequency, frequency-domain exploration. It aimed to enhance operational flexibility via a rigid-boom pod configuration. However, its utility in high-interference urban settings was limited by its low signal-to-noise ratio (SNR) and low transmitter power [86].

**Figure 5:**
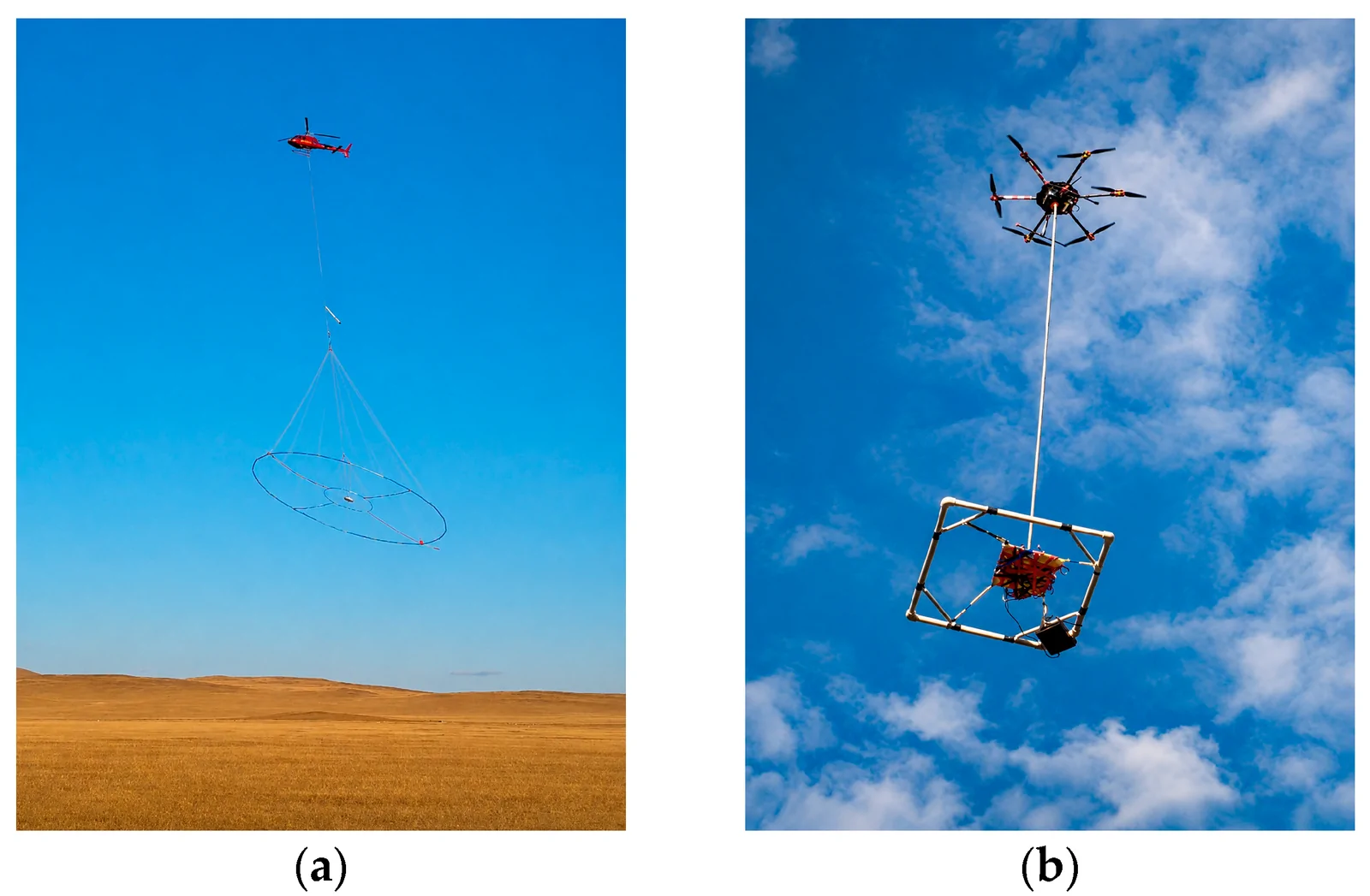
Representative airborne TEM sensing platforms. (**a**) CAS-HTEM helicopter-borne TEM system, reproduced from Ref. [26], which is published under the Creative Commons Attribution 4.0 License. (**b**) SibGIS UAV-based electromagnetic system, reproduced from Ref. [24], an open-access article published in Applied Sciences, in accordance with the applicable Creative Commons license.

The China Aero Geophysical Survey and Remote Sensing Centre for Land and Resources (AGRS), in collaboration with Jilin University and other institutions, developed the CHTEM-I system. The system has a rigid frame and an eccentric buckling configuration. It utilizes a transmitter loop with a radius of 6 m, a peak transmitter current of 450–500 A, a magnetic moment of approximately 0.26 MA·m^2^, and a turn-off time of around 1 millisecond [79]. It is primarily suitable for medium-depth mineral prospecting. The upgraded CHTEM-II system then advanced the transmitter magnetic moment to 0.354 MA·m^2^ and incorporated a single-degree-of-freedom mechanically resonant receiver assembly, suppressing the dynamic noise floor to 5 nT/s [23]. Furthermore, the Chinese Academy of Sciences (CAS) developed the CAS-HTEM system in cooperation with Jilin University (Figure 5a). This system adopts a flexible frame and a central-loop configuration. Scaling the transmitter magnetic moment to approximately 0.675 MA·m^2^, compressing the turn-off time to 450 μs, and equipping the platform with a three-axis induction magnetometer and a dual-gain-channel receiver enabled the system to achieve a low noise floor of 0.1 nT/s. This successfully balances deep-seated penetration with shallow, high-resolution imaging capabilities [26].

The increasing use of uncrewed aerial vehicle (UAV) platforms for low-altitude sensing has brought airborne electromagnetics into the realm of ultra-shallow, high-resolution detection. Parshin et al. [24] pointed out that the paramount advantage of UAV platforms lies in their exceptional terrain-following capability. By keeping the sensors at an altitude of just 10–20 m above the ground, high signal-to-noise ratio (SNR) data can be acquired even with low-power transmitters (Figure 5b). To adapt to complex urban conditions, domestic researchers have utilized carbon fiber composite materials and lightweight integration technologies to successfully develop fully integrated UAV pods weighing less than 50 kg [23]. Teams from Jilin University and other institutions have conducted shallow exploration trials using multi-rotor UAVs equipped with TEM systems, demonstrating the potential to rapidly screen for urban road cave-in hazards. Recent lightweight UAV-TEM systems with integrated acquisition-to-interpretation workflows have further validated continuous mobile measurement and automatic target identification from multi-rotor platforms [80], and drone-borne electromagnetic surveys have been cross-validated against ground truth in field campaigns [81].

To strike a balance between exploration depth and operational flexibility, the semi-airborne transient electromagnetic (semi-airborne TEM) modality provides an effective solution. Wu et al. [27] reviewed the evolution of semi-airborne platforms in China, in which the transmitter system is deployed on the ground, and lightweight sensors are flown via uncrewed aerial vehicles (UAVs) to record signals in the air. This ‘ground-air asymmetric’ configuration relaxes the payload limitations of airborne carriers while maintaining the ability to conduct deep sounding. Meanwhile, the Chinese Academy of Sciences used a modified large single-rotor UAV to conduct three-component magnetic field measurements in an iron-mining area in Shandong Province. The resulting inversion profiles correlated exceptionally well with existing geological boreholes. Furthermore, Shandong University developed a lightweight semi-airborne transient electromagnetic receiver system, SATEM, weighing less than 3 kg, with a receiver coil diameter of 0.5 m, an effective area of approximately 10 m^2^, a sampling rate of 256 kHz, and a bandwidth of around 56 kHz. This system uses GNSS second-pulse outputs for synchronization [82]. Becken [83] also adopted a semi-airborne architecture for the DESMEX system, deploying components such as fiber-optic laser gyroscopes to measure pod attitude in real time, thereby ensuring the spatial orientation accuracy of the recorded magnetic field components. Semi-airborne configurations have likewise been demonstrated for exploring shallow landslide structures under complex surface conditions [84].

By systematically contrasting the technical specifications of current mainstream platforms (Table 6), the evolutionary trajectories of contemporary research become evident: shifting from the early-stage paradigm of SkyTEM, which pursued massive magnetic moments for deep prospecting, toward modern objectives characterized by minimized blind zones, high dynamic adaptability, ground-air cooperation, and multi-scale detection. Future airborne platforms will no longer operate as isolated entities but will instead evolve toward sensor arrays and multi-UAV collaborative networks. This transition from standalone carrier technology to collective swarm-intelligence sensing will be the cornerstone of realizing fine-scale imaging of underground spaces in highly complex urban environments.

**Table 6:** Comparison of key technical parameters of typical airborne and UAV sensing platforms.

Name	Developer/Source	Configuration	Transmitter Magnetic Moment (A·m^2^)	Turn-Off Time (μs)	Detection Depth (m)
SkyTEM	Aarhus University, Aarhus, Denmark	Helicopter-borne rigid pod	Dual-moment mode: 3.5 k/250 k	12	0–350
AeroTEM	Aeroquest, Mississauga, ON, Canada	Helicopter-borne flexible-link pod	~40 k	<20	200–300
HeliTEM	CGG, Massy, France	Helicopter-borne flexible-link pod	~2 M	~200	>500
CHTEM-I	AGRS/Jilin University, Changchun, Jilin, China	Helicopter-borne rigid pod	~0.26 M	~1000	200–300
CAS-HTEM	CAS/Jilin University, Changchun, Jilin, China	Helicopter-borne flexible frame (Central loop)	~0.675 M	450	>600
SAEM	CAS/Jilin University, Changchun, Jilin, China	Ground transmitter + UAV/Airship receiver	~500 k	Dynamically adjustable via ground Tx (typically long)	300–1000
SATEM	Shandong University, Jinan, Shandong, China	Ground transmitter + UAV receiver	Dependent on the ground source	~50	>200

Notes: Magnetic-moment values are peak nominal figures reported by the developers; dual-moment systems list both low-moment and high-moment settings. Detection depths are nominal maxima under favorable geological and noise conditions and can degrade substantially in the presence of strong urban interference; ‘~’ denotes approximate values.

### 3.3. Field Experiments

(1) Ground Mobile Platforms

To validate the reliability and speed of survey results obtained using ground-mobile transient electromagnetic (TEM) sensing platforms in complex urban environments, An et al. from Jilin University conducted field experiments along urban roads and in subway construction zones in Changchun using their self-developed rTEM system [57]. The survey area consisted of asphalt pavement adjacent to green belts, with complex subsurface structures including subway stations and shield tunnels. Multiple parallel survey lines were acquired, and three-dimensional resistivity imaging was performed to characterize shallow geological structures, subway facilities, and groundwater accumulation zones. The results demonstrated that the subway structures generated distinct resistivity responses, while localized low-resistivity anomalies were associated with groundwater infiltration and accumulation. These experiments confirmed the capability of ground-mobile TEM platforms for continuous urban infrastructure monitoring and subsurface hazard identification under traffic conditions.

**Figure 6:**
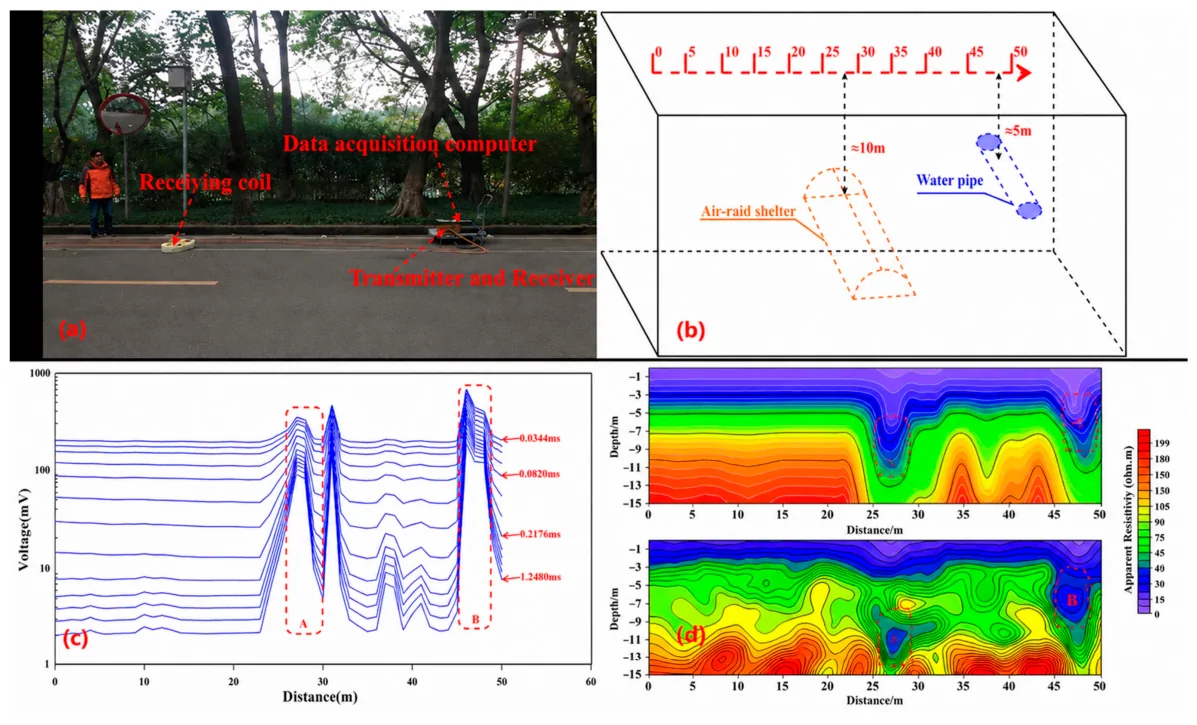
Field configuration, measured TEM responses, and imaging results at the air-raid shelter site of Chongqing University. (**a**) Field acquisition configuration showing the receiving coil, transmitter–receiver assembly, and data-acquisition unit. (**b**) Schematic representation of the survey line and the known subsurface targets, including an air-raid shelter at a depth of approximately 10 m and a water pipe at a depth of approximately 5 m. (**c**) Measured multi-time-channel voltage profiles along the survey line, with the two principal anomalous zones marked as A and B. (**d**) Apparent-resistivity sections obtained using conventional “smoke-ring” rapid imaging (upper panel) and particle swarm optimization (PSO) inversion (lower panel); anomalies A and B correspond to the air-raid shelter and water pipe, respectively. Reproduced from Ref. [87], which is published under the Creative Commons Attribution 4.0 License.

In addition to continuous profiling applications, high-precision inversion of ground-based TEM measurements has also been demonstrated in urban engineering investigations. Xu et al. [87] conducted a field experiment at the air-raid shelter site of Chongqing University using the FCTEM60-1 transient electromagnetic system. As shown in Figure 6a, the field acquisition system consisted of the transmitter–receiver assembly, the receiving coil, and the data-acquisition unit. Figure 6b illustrates the survey geometry and the locations of two representative subsurface targets along the approximately 50 m survey line: an air-raid shelter at a burial depth of approximately 10 m and a water pipe at a depth of approximately 5 m. The measured multi-time-channel voltage responses are presented in Figure 6c, where distinct anomalies occur near the horizontal positions of the two known targets. Figure 6d further compares the apparent-resistivity sections obtained using conventional “smoke-ring” rapid imaging and particle swarm optimization (PSO) inversion. Both methods identify the principal anomalous zones, whereas the PSO inversion provides a more detailed representation of their spatial distribution and boundaries. In particular, the anomalies located at approximately 27 m and 47 m along the profile correspond well to the known air-raid shelter and water pipe, respectively. These results demonstrate that nonlinear inversion can improve the interpretation of small-scale underground structures and low-resistivity anomalies in complex urban environments.

This field experiment provides direct evidence that advanced inversion algorithms can significantly enhance the interpretation capability of ground-mobile TEM platforms in urban environments. By combining reliable transient-response acquisition with nonlinear-optimization-based inversion, ground TEM systems can achieve improved delineation of small-scale underground structures and low-resistivity hazards, thereby enabling more refined detection in urban underground spaces.

(2) Airborne Platforms

To simulate potential zones of ground collapse, Chang’an University constructed a three-dimensional (3D) geological model of a typical city in eastern China. The model was embedded with four subsurface hydraulic connection channels. A numerical simulation experiment was then conducted using a grounded-source semi-airborne time-domain electromagnetic (TEM) configuration. The transmitter source, with a wire length of 1000 m, was deployed flexibly on the ground in areas with favorable grounding conditions, such as garden flowerbeds. Meanwhile, the receiver system was carried by a UAV at an altitude of 50 m, covering a transient measurement window from 1 μs to 6 ms. Full-area apparent resistivity imaging results show that the locations of the low-resistivity channels closely match the predefined model and that the imaging boundaries are clearly defined [88]. This numerical trial robustly demonstrates that, even in complex urban environments characterized by densely populated high-rise buildings and constrained ground conditions, the semi-airborne TEM system can flexibly deploy its transmitter on the ground and record signals via an airborne UAV. This configuration successfully detects hidden subsurface hazards, providing critical technical support for the early warning of urban ground collapses.

**Figure 7:**
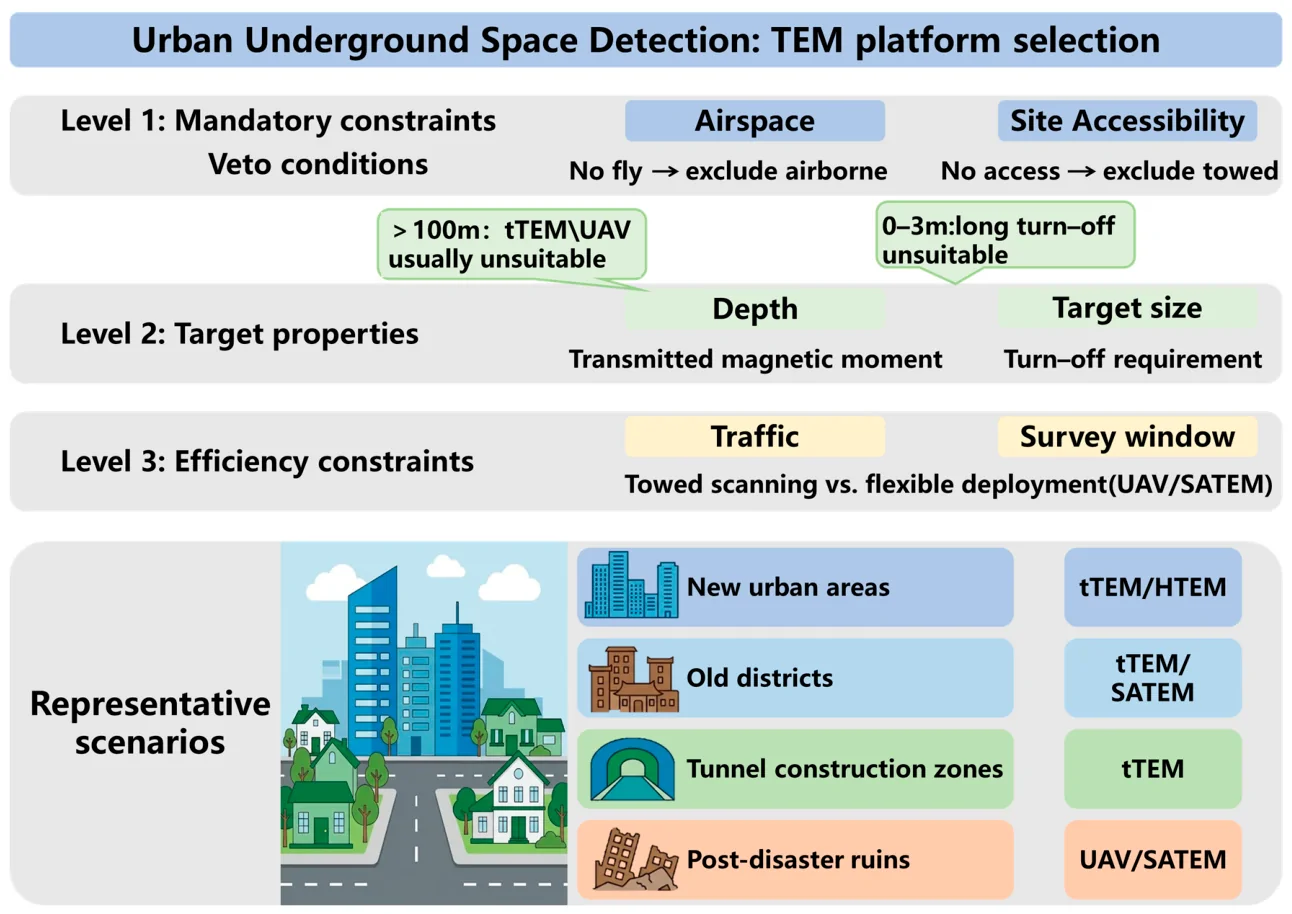
Decision framework for selecting TEM sensing platforms for urban underground-space detection. Level 1 contains mandatory constraints: airspace restrictions can exclude airborne platforms, whereas insufficient ground accessibility can exclude towed platforms. Level 2 evaluates target properties, including burial depth and target size, which determine the required transmitter magnetic moment and turn-off performance. Level 3 evaluates operational-efficiency constraints, including traffic conditions and the available survey window. Based on these factors, representative platform recommendations are provided for newly developed urban areas, old districts with dense infrastructure, tunnel-construction zones, and post-disaster or restricted-access areas. tTEM denotes ground-towed TEM; HTEM denotes helicopter-borne TEM; UAV-TEM denotes UAV-borne TEM; and SATEM denotes semi-airborne TEM. The depth thresholds shown are indicative values derived from the nominal platform envelopes summarized in Table 5 and Table 6 rather than absolute limits.

The aforementioned field experiments effectively demonstrate the distinctive detection capabilities of ground-towed and semi-airborne TEM systems in various urban environments. However, in practical urban underground space exploration, factors such as target depth, site openness, airspace restrictions, and traffic conditions vary drastically, meaning that each platform has its own optimal deployment niche. To provide intuitive guidance for platform selection, Figure 7 presents a TEM platform selection decision framework whose criteria are derived from the parameter envelopes in Table 5 and Table 6 and from the scenario-applicability analysis in Section 3.4. The decision logic assigns different weights to three categories of constraint factors: (i) mandatory constraints-airspace control and site accessibility-which act as veto conditions that directly exclude airborne or ground options regardless of their technical merits; (ii) target attributes-burial depth and expected target size-which determine the required transmitter magnetic moment and turn-off performance, and hence the feasible platform family (for example, targets deeper than about 100 m essentially exclude lightweight UAV-borne systems, whereas ultra-shallow 0–3 m cavities exclude platforms with long turn-off times); and (iii) efficiency constraints-traffic conditions and the available survey window-which decide whether continuous towed scanning, rapid airborne reconnaissance or flexible UAV/semi-airborne deployment is preferable. For refined working conditions, newly built urban districts with open spaces and favorable operational conditions may accommodate both towed and helicopter-borne systems: towed TEM is preferred for high-resolution continuous profiling along accessible roads, whereas helicopter-borne TEM is advantageous for rapid regional reconnaissance owing to its high acquisition efficiency and broad spatial coverage. Therefore, the selection between ground-towed and helicopter-borne TEM in newly built districts depends mainly on whether the priority is detailed engineering investigation or large-area rapid screening. Old districts with dense high-rises and overlapping metallic pipelines favor small-loop ground systems with structural decoupling and active denoising, or semi-airborne configurations where a grounded transmitter site is available; tunnel construction zones and underpasses, where both airspace and surface space are restricted, favor vehicle-mounted small-loop (e.g., OCTEM-type) systems; and post-disaster ruins or restricted-access areas favor UAV-borne or semi-airborne platforms. The boundary values of the decision framework (e.g., the depth thresholds between platform families) correspond to the nominal detection-depth ranges in Table 5 and Table 6 and should be interpreted as typical values under favorable conditions rather than absolute limits.

### 3.4. Applicability and Limitations of Sensing Platforms in Typical Urban Scenarios

Although the platform families reviewed above are often presented as general-purpose solutions, their practical performance in cities is strongly scenario-dependent. Table 7 summarizes, in a unified manner, the applicability and inherent limitations of the four platform families in four typical urban working conditions, consolidating scenario-specific limitations that are otherwise scattered across the literature. Several cross-cutting limitations deserve emphasis.

**Table 7:** Applicability and limitations of TEM platforms under representative urban scenarios.

Urban Scenario	Ground-Towed	Helicopter-Borne	UAV-Borne	Semi-Airborne
Newly built districts with open paved roads	*** High-efficiency continuous profiling; preferred for detailed engineering investigation; traffic coordination required	*** High-efficiency regional reconnaissance	** Flexible but lower areal efficiency than towed systems	** Requires a grounding site; slower deployment
Old districts with dense pipelines	** Small-loop plus decoupling viable; metal coupling masks anomalies; congestion limits speed	* Shallow blind zone; building-induced flight restrictions	** Low-altitude flight constrained by buildings; metal coupling remains	*** Ground transmitter in available greenbelts and UAV receiver bypass congestion; grounding may be poor
Tunnel construction zones	** Vehicle-mounted systems along access roads; vibration and metal-support interference	* Restricted by flight permits over active sites	** Limited by permits and site safety	** Useful for advance detection around portals and access areas
Post-disaster ruins/restricted-access areas	* Ground access impossible	** Rapid large-area reconnaissance; high cost; shallow blind zone	*** Non-contact, terrain-independent; limited depth (typically <50 m)	*** Deep sounding without ground access; at least one grounding site required

Notes: *** denotes preferred applicability, ** denotes applicability subject to operational or environmental constraints, and * denotes generally unsuitable conditions. These ratings represent a qualitative synthesis of the platform capabilities, nominal parameter envelopes in Table 5 and Table 6, and scenario-specific limitations reported in the cited literature; they are not derived from a unified experimental benchmark.

First, in newly built districts with open paved roads, platform selection mainly depends on the balance between investigation objectives and survey efficiency. Towed TEM systems are advantageous for high-resolution continuous profiling and detailed engineering investigations. In contrast, helicopter-borne TEM systems provide rapid regional reconnaissance capability due to their extensive spatial coverage and high acquisition efficiency. However, the latter remains unsuitable for ultra-shallow targets due to its relatively long turn-off time. UAV-borne and semi-airborne systems offer flexible deployment but are constrained by payload capacity or transmitter-site requirements.

Second, in old districts with dense pipelines, coupling responses from urban infrastructure may mask or distort genuine anomalies for all platform families. Small-loop tTEM systems with structural decoupling and active denoising offer greater adaptability in such environments. At the same time, semi-airborne configurations can be considered when suitable ground-based transmitter sites are available. Nevertheless, complete separation of infrastructure-induced responses remains challenging.

Third, in tunnel construction zones, the applicability of TEM platforms is primarily determined by access conditions, safety requirements, and the availability of suitable survey spaces. Vehicle-mounted systems can operate along accessible construction roads but may be affected by vibration and metal-to-metal interference. UAV-borne and semi-airborne systems provide non-contact detection capability around portals and restricted areas but remain constrained by flight regulations and transmitter deployment conditions.

Fourth, in post-disaster ruins or restricted-access areas, conventional ground platforms may become unavailable because of blocked roads and unsafe conditions. UAV-borne and semi-airborne systems therefore provide important alternatives for rapid reconnaissance and investigation of inaccessible areas, although their limited transmitter power may limit deep exploration capability.

## 4. Discussion

**Figure 8:**
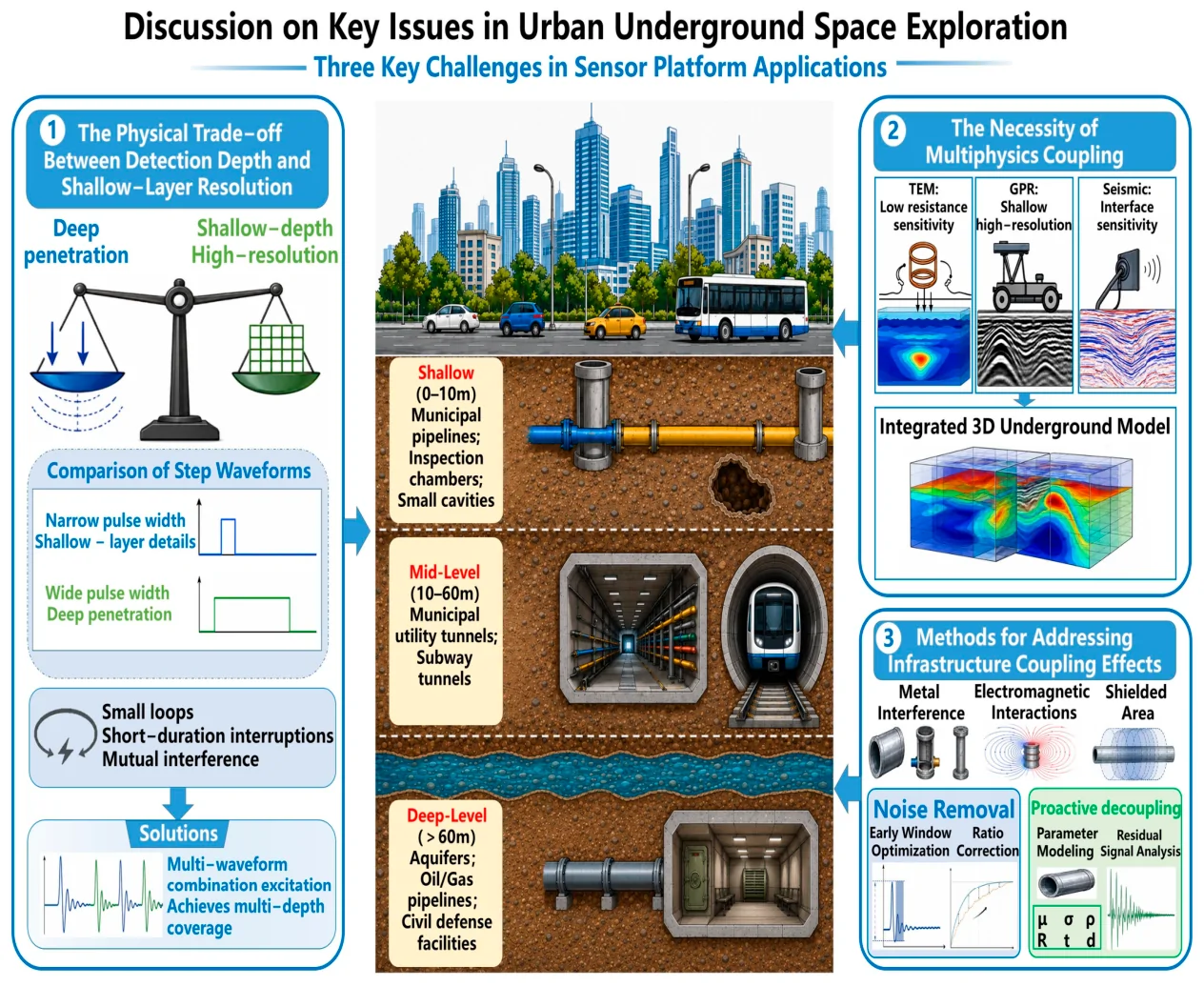
Schematic summary of key issues in sensor platform applications for urban underground space exploration.

Due to the complex nature of urban underground space exploration, including highly intricate targets, significant variations in burial depths, dense existing infrastructure, and overlapping anomalies, sensing platforms must not only ensure adequate penetration depth but also balance resolving shallow targets with reliably interpreting data in confined spaces. Consequently, this section outlines the discussion of three key issues in urban underground space exploration: (1) the physical trade-off between detection depth and shallow resolution; (2) the necessity of multi-physics fusion for identifying complex subsurface structures; and (3) coupling interference induced by existing urban infrastructure and its mitigation methodologies. These three interconnected issues collectively dictate the detection precision, interpretation reliability, and engineering applicability of sensor platforms in urban scenarios, as illustrated in Figure 8.

(1) Physical Trade-off Between Detection Depth and Shallow Resolution

As shown on the left of Figure 8, there is a clear trade-off between detection depth and shallow resolution when exploring urban underground spaces. To characterize shallow anomalies during urban surveys, the transmitter current must terminate within an ultra-short time window. While small-loop systems can overcome spatial footprint constraints, the resulting mutual inductance interference severely limits shallow resolution, creating an inherent physical bottleneck [35]. Traditional single-waveform excitation paradigms typically fail to simultaneously reconcile large-depth energy penetration with high-frequency shallow details. To overcome this issue, multi-waveform composite excitation strategies have emerged as a vital technical development in recent years [37,38]. Based on a comprehensive review of existing methodologies, the authors contend that the future breakthrough lies in a ‘multi-waveform combined excitation’ strategy. This approach uses the high-frequency components of narrow-pulse currents to detect subtle hazards at depths of a few meters. In contrast, the high energy of wide-pulse currents penetrates deep utility tunnels. This methodology compensates for deficiencies in physical sensor dimensions via time-sequence combinations and constitutes a critical pathway towards achieving full-depth subsurface coverage.

(2) The Necessity of Multi-Physics Fusion Technologies

As shown in the top right of Figure 8, the variety of target types in complex urban underground spaces means that it is rarely possible for a single geophysical method to meet the concurrent requirements of fine-scale shallow identification, deep anomaly sounding, and structural boundary delineation [89]. This discussion asserts that multi-physics fusion is now a core configuration, rather than merely a supplementary tool. The co-platform integration of transient electromagnetics with seismic/vibrational sensing or ground-penetrating radar (GPR) can significantly mitigate the inherent limitations of TEM in specific scenarios by exploiting the sensitivity of seismic waves to medium interfaces and voids, or the high-resolution imaging capability of GPR in the near-surface [90]. Furthermore, collaborative inversion and joint constraints across multiple geophysical datasets have been shown to effectively improve the reliability of subsurface geoelectrical models [91,92]. Since electromagnetic fields are sensitive to low-resistivity features and seismic waves respond to structural interfaces and cavities, spatially constrained imaging using multi-field data can greatly improve the accuracy of early warnings for road cave-in hazards and achieve fine-scale characterization of lithological and structural attributes [93].

(3) Treatment Methodologies for Infrastructure Coupling Effects

As shown in the lower-right quadrant of Figure 8, existing infrastructure widely distributed throughout urban underground spaces—including metallic pipelines, maintenance holes, shoring structures, subway tunnels, and comprehensive utility corridors—exerts pronounced coupling effects on electromagnetic wave propagation and the resulting anomalous responses [43]. To address this challenge, two distinct philosophies have emerged in contemporary research. The first treats these effects strictly as noise to be eliminated. Capitalizing on the fact that metallic interference is predominantly concentrated in early-time windows, researchers separate the true anomalous response from the interference signals by optimizing observation time windows or implementing ratio-correction algorithms [94]. However, simply discarding these data risks obliterating critical geological information. Conversely, the second philosophy advocates treating these responses as tracer signals for pipeline localization. By explicitly incorporating pipeline geometric and physical parameters into the forward model, precise signal decoupling can be achieved [41,56]. This strategy not only accurately localizes the infrastructure itself but also enables the rigorous analysis of residual signals to determine whether geomechanical looseness or hidden cavities exist around the pipelines. This profound paradigm shift from passive denoising to active decoupling is the cornerstone for resolving strong-coupling interference challenges in complex urban environments.

While the three pathways above are conceptually clear, their engineering implementation is constrained by practical bottlenecks that warrant explicit recognition. For multi-waveform composite excitation, simultaneously widening the spectrum and increasing the transmitted energy raises the peak power, thermal load, and battery demand of the transmitter, which directly conflicts with the payload and endurance limits of UAV-borne platforms; waveform-programmable, high-efficiency transmitter topologies (e.g., RLC-resonant inverters [40]) only partially relieve this conflict. For multi-physics fusion, heterogeneous sensors differ in spatial sampling interval, positioning accuracy, and acquisition rhythm, so multi-source data registration and time synchronization remain error-prone on moving platforms, and joint forward/inversion models typically require one to two orders of magnitude more computation than single-physics inversion, which is difficult to sustain for city-scale datasets. For active decoupling of pipeline coupling interference, accurate a priori knowledge of pipeline geometry, burial depth, and material is required, whereas urban pipeline archives are frequently incomplete or outdated. Moreover, synchronous multi-channel acquisition hardware that records strong pipeline responses and weak geological responses without saturation remains limited by receiver dynamic range. These bottlenecks indicate that near-term progress depends as much on hardware engineering—transmitter efficiency, receiver dynamic range, and synchronization—as on algorithmic innovation.

## 5. Conclusions and Outlook

### 5.1. Conclusions

Through a comprehensive review of the state-of-the-art in transient electromagnetic (TEM) sensing platform technologies tailored for urban underground spaces, the primary conclusions are derived as follows:

(1) Substantial Advancements in Sensor Hardware Performance: By implementing high-frequency fast turn-off circuits and non-coplanar bucking configurations, contemporary TEM systems successfully suppress system mutual inductance and drastically compress early-time blind zones. Although constrained by inherent physical boundaries and not fully eliminated, these configurations have enabled high-precision scanning of near-surface hazards beneath urban roads, laying a robust physical foundation for fine-scale subsurface exploration.

(2) Paradigm Shift in Interference Mitigation from Passive to Active: The methodology for noise suppression in urban settings no longer relies solely on post-processing digital filtering. Leveraging hardware-level online calibration, mechanical vibration-reduction architectures, and deep learning algorithms, modern sensing platforms can accurately extract weak geoelectrical echo signals from complex urban power-line and cultural background noise.

(3) Diversification of Carrier Platforms and Operational Modalities: A range of mobile sensing platforms has been developed to accommodate the spatial and operational constraints of urban environments, including ground/towed systems, UAV-borne platforms, and semi-airborne configurations. These platforms provide different levels of operational flexibility while balancing detection depth, spatial resolution, survey efficiency, and deployment constraints.

(4) Advances in Imaging and Inversion Algorithms: Virtual wave-field migration, multi-resolution imaging, and learning-based rapid inversion have expanded TEM interpretation from conventional apparent-resistivity mapping toward higher-resolution and more computationally efficient imaging. These approaches improve the delineation of complex subsurface structures and provide algorithmic support for near-real-time processing of continuously acquired TEM data.

### 5.2. Outlook

Looking forward, urban transient electromagnetic exploration is poised to converge toward three primary frontiers: fully autonomous platforms, multi-element data fusion, and fully transparent real-time early warnings.

(1) Fully Autonomous Intelligent Platforms: Sensing carriers will be endowed with higher levels of autonomous trajectory planning, fostering seamless cooperative swarms between uncrewed ground vehicles (UGVs) and UAVs. The main technical bottlenecks are microsecond-to-millisecond time synchronization between swarm nodes under GNSS-denied conditions (e.g., beneath overpasses and in underpasses), autonomous obstacle avoidance and formation keeping in obstacle-dense urban airspace subject to strict flight regulations, and distributed task allocation that balances coverage, endurance, and data quality. Feasible breakthrough paths include GNSS/INS/UWB fused relative positioning, synchronization disciplined by GNSS pulse-per-second signals with high-stability oscillator holdover [82], and hierarchical swarm architectures in which a semi-airborne controller node provides deep sounding. In contrast, lightweight UAV agent nodes perform fine-scale resurveying. Through multi-node, heterogeneous network observations, these systems will overcome the challenge of holistic information acquisition beneath complex, disrupted urban surfaces.

(2) Multi-Element Data Fusion: Future processing frameworks are expected to increasingly incorporate joint inversion of multiple geophysical fields, integrating electrical, magnetic, mechanical, and acoustic information. The key bottlenecks are the registration and gridding of heterogeneous, multi-source data with different footprints and resolutions, the scarcity of petrophysical relationships linking distinct physical properties, and the high computational cost of joint 3-D forward and inversion models. Promising breakthrough paths include structurally coupled joint inversion with cross-gradient constraints [89,91], physics-guided deep learning-based surrogate forward models [75] that reduce joint inversion costs by orders of magnitude, and edge-cloud collaborative computing, in which edge terminals perform fast screening. In contrast, the cloud performs full joint inversion. By harnessing the complementary constraints imposed by distinct physical properties, this multi-field synergy will substantially alleviate the non-uniqueness inherent in standalone geophysical modalities, ultimately enabling the construction of high-fidelity three-dimensional (3-D) subsurface geoelectrical models.

(3) Real-Time Transparent Early Warnings: Powered by high-speed edge computing terminals and cloud computing architectures, next-generation TEM systems will unlock online concurrent imaging and automated hazard identification. The principal bottlenecks are lightweight inversion engines that fit the power and memory budgets of vehicle- or UAV-mounted edge hardware; the real-time transmission of heterogeneous, multi-source data under limited urban communication bandwidth; and automated quality control that prevents noise-driven false alarms during unsupervised operation. Feasible breakthrough paths include compressed acquisition and event-triggered data reduction, quantized or pruned neural inversion surrogates deployable on embedded GPU/FPGA hardware [73,75], and 5G edge-cloud pipelines that transmit anomaly-level products instead of raw waveforms. This scanning-to-imaging dynamic monitoring capability will transform TEM technology from a standalone prospecting tool into an embedded digital sensor for the structural health monitoring of urban infrastructure, extending a solid technological cornerstone for the construction of resilient cities.

## Figures and Tables

**Table 1:** Comparison of mainstream methods for underground space detection.

Geophysical Methods	Depth of Detection	Sensitivity to Low Resistance	Efficiency	Suitability for Urban Environment
Electrical resistivity tomography (ERT)	0–100 m	**	*	Inapplicable to urban paved roads
Seismic exploration	0–200 m	*	*	Requires seismic sources and geophone arrays; difficult to deploy in urban areas.
Ground-penetrating radar (GPR)	0–20 m	**	***	Limited depth; severe attenuation in high-conductivity media
Transient Electromagnetic (TEM)	0–500 m	***	***	Adaptable to paved roads and complex electromagnetic environments

Notes: The symbols represent the relative applicability of each TEM platform under the corresponding conditions. *** indicates high applicability or preferred suitability; ** indicates moderate applicability subject to operational or environmental constraints; * indicates limited applicability or generally unsuitable conditions. These qualitative ratings are synthesized from reported platform capabilities, operational constraints, and representative application scenarios in the cited literature, rather than from a unified experimental benchmark.

## Data Availability

No new data were created or analyzed in this study. Data sharing is not applicable to this article.
